# Healthy working time arrangements for healthcare personnel and patients: a systematic literature review

**DOI:** 10.1186/s12913-019-3993-5

**Published:** 2019-03-27

**Authors:** Vilde H. Bernstrøm, Daniele Evelin Alves, Dag Ellingsen, Mari Holm Ingelsrud

**Affiliations:** OsloMet – Oslo Metropolitan University, Work Research Institute, P.O.Box 4 St. Olavs Plass, N-0130 OSLO, Oslo, Norway

**Keywords:** Working time, Shift work, Health, Patient safety, Health sector, Systematic review

## Abstract

****Background**:**

A number of working time arrangements have been linked to negative consequences for both health personnel and their patients. A common hypothesis put forth to explain these findings suggests that certain working time arrangements lead to negative patient consequences due to the adverse impact they have on employee health. The purpose of this study is to use systematic reviews to investigate whether employee health explains the relationship between working time arrangements and patient safety.

****Methods**:**

A systematic literature review was performed including published reviews and original studies from MEDLINE, PsycINFO, Cinahl and Web of Science investigating working time arrangements for healthcare personnel, employee health and patient safety. In addition, we screened reference lists of identified reviews. Two reviewers independently identified relevant publications according to inclusion criteria, extracted findings and assessed quality.

****Results**:**

Six thousand nine hundred thirty papers were identified, of which 52 studies met our criteria. Articles were categorized into five groups according to how they approached the research question: 1) independent analyses of relationship between working time arrangements and employee health, and of working time arrangements and patient safety (5 studies); 2) relationship between working time arrangements on *both* employee health and patient safety (21 studies); 3) working time arrangements and employee health as two explanatory variables for patient safety (8 studies); 4) combinations of the above analyses (7 studies); 5) other relevant studies (5 studies). Studies that find that working time is detrimental to employee health, generally also find detrimental results for patient safety. This is particularly shown through increases in errors by health personnel. When controlling for employee health, the relationship between working time arrangements and patient safety is reduced, but still significant.

****Conclusions**:**

Results suggest that employee health partially (but not completely) mediates the relationship between working time arrangements and patient safety. However, there is a lack of studies directly investigating employee health as a mediator between working time arrangements and patient safety. Future studies should address this research gap.

**Electronic supplementary material:**

The online version of this article (10.1186/s12913-019-3993-5) contains supplementary material, which is available to authorized users.

## Background

Studies have shown negative health consequences from shift work and other atypical working time arrangements [1, 2]. Within the health sector, several of the working time arrangements that adversely affect employees’ health also correlate with negative patient safety consequences [1, 2]. The objective of this systematic literature review is to investigate whether the relationship between working time arrangements and patient safety is explained (or mediated) by employee health.

For example, shift length, and particularly shifts exceeding 10–12 h, has been linked to negative health effects among staff, such as reduced mental health [3, 4], sleepiness and fatigue [1], and overweight [5]. At the same time, increased shift length has been tied to negative patient consequences, such as higher risk of error among nurses and nurses’ aides [1, 6]. Similarly, studies have indicated that night work is related to negative consequences among employees, such as reduced quality of sleep [2], increased risk of depression [7, 8], and heart and vascular diseases [9], in addition to higher risk of error among health staff and increased mortality among patients at night [2, 10].

Researchers have associated the adverse employee health consequences with negative consequences for patient safety [3, 6]. Their hypothesis is that working time arrangements that lead to reduced sleep, increased fatigue and decreased alertness among staff also lead to reduced patient safety [3, 6]. That employee health can mediate the correlation between working time arrangements and patient safety is supported by studies showing that sleep disruption and limited sleep diminish the ability to carry out cognitive tasks [11]. Emotional fatigue has also been tied to needle stick injuries [12], and burnout among nurses has been connected to reduced patient satisfaction [13]. Additionally, longer working days have been linked to fatigue among nurses, which in turn has been tied to lower self-assessments of job performance [14].

Even though certain working time arrangements that are detrimental to employee health also seem to be detrimental to patient safety, there may be cases when measures promoting aspects of patient safety conflict with those promoting employee health. Introducing healthy working time arrangements among health personnel may lead to unfortunate consequences for patient safety, such as more frequent changes in attending doctors [15]. Some studies have also supported the idea that multiple night shifts in a row can raise the quality of patient care while simultaneously increasing the risk of breast cancer among staff [3, 4]. Furthermore, while several studies have pointed out the negative consequences of extended shifts for both patients and staff, not all studies concur [16]. Other factors likely affect the correlation, such as amount of rest during the shift and the total number of working hours per week [17–19].

Consistency among studies assessing consequences of working time arrangements for employee health and patient safety, and more knowledge on causality, are important factors to investigate. Such research may potentially suggest that introducing healthy working time arrangements may result in a win-win situation for both staff, in terms of health, and patients, in terms of safety. If the correlation between working time and patient safety is mediated by employee health, measures to improve employee health will be important for patient safety as well.

The objective of this systematic literature review is to investigate whether the relationship between working time arrangements and patient safety is explained (or mediated) by employee health. This systematic literature review will include studies that directly aim to test whether employee health mediates (accounts for) the relationship between working time arrangements and patient safety. The review will also include empirical studies investigating the consequences of working time arrangements for both employee health and patient safety within the same study. As the number of studies directly testing mediation is low, we also include studies that provide indirect information about mediation.

For instance, an indirect indication of mediation would be controlling for employee health in a regression model assessing the relationship between working time arrangements and patient safety. If the correlation between working time arrangements and patient safety disappears (or is significantly reduced) after controlling for employee health in a statistical model, there is indication of full- (or partial mediation, respectively). In contrast, if multiple studies find a relationship between working time arrangements and patient safety after controlling for employee health, employee health is unlikely to fully mediate the relationship.

Another indication of the mediation of employee health between working time arrangements and patient safety would be studies that investigate how working time arrangements relate to employee health and patient safety as two separate outcomes. If employee health completely mediates the relationship between working time arrangements and patient safety, working time arrangements that are unrelated to employee health should also be unrelated to patient safety. If employee health only partially mediates the relationship, we would still expect to more often find a correlation in the same direction between working time arrangements and *both* patient safety and employee health. A final indication of mediation can be found in studies in which working time arrangements are related to employee health, and employee health is related to patient safety.

Having the above indications of mediation in mind, we pose the following research question in this systematic review: Is the relationship between working time arrangements and patient safety explained by employee health?

## Method

A literature review protocol was developed to design search strategies, selection criteria, type of measures included and quality assessment criteria. We used the PRISMA 2009 checklist (Preferred Items for Systematic Reviews and Meta-Analyses) to report this review.

### Search process

We performed a systematic search in Medline, PsychInfo, Web of Science and Cinahl, and screened previous literature reviews (e.g. [1, 6, 10]) to ensure the inclusion of relevant papers from their reference lists. The final search, carried out in January 2018, included key words and topic words connected to working time arrangements *and* patient safety *and* health sector personnel/employee health. We limited the search by language (see Selection criteria). The final search, adapted to all databases, is included in Additional file 1: Appendix 1.

### Selection process

All titles and abstracts were independently evaluated by two experienced researchers based on the selection criteria. Screening was conducted in Covidence [20] following two consecutive steps: (1) screening based on title and abstract, and, if selection criteria were met, (2) screening based on full text. Disagreements were discussed until the researchers reached agreement. In cases of uncertainty, the article was examined in its entirety.

### Selection criteria

#### We included studies that fulfilled the following criteria

The article analyzes the correlation between working time arrangements, employee health *and* patient safety with data from the health sector. Working time arrangements are all measures of when and how long employees work (e.g., hours per week, hours per shift, evening work, night work, rotating shift work). Employee health is broadly defined and includes illness, injury, sleep problems and other physical or mental health issues. Patient safety is defined as medical outcome measures for patients (e.g., mortality, infections, wounds), and errors made in patient care.

Only empirically original studies published in peer-reviewed journals, written in English, Norwegian, Swedish or Danish were included. There were no limitations on the year of publication.

We excluded articles that only indirectly investigate patient safety, for instance through indicators of patient safety (such as healthcare employees’ performance and cognitive abilities), or through other patient outcomes besides safety (such as patient satisfaction). Exceptions were studies using simulated tests that closely mimicked patient care (e.g., if erroneous treatment in a simulated operating room was included, rather than general ability tests for reaction time and memory). We also excluded articles focusing on the 2003 working time reform in the USA, not based on year restrictions, but based on their relevance to our research question in terms of their description of a specific intervention at the extreme end in terms of working hours. These studies have been thoroughly covered in several previous literature reviews with a different focus than the current review [15, 21, 22].

### Data extraction and quality assessment

Data extracted from the included papers was entered into a standardized spreadsheet (including authors, publication year, operationalization of key variables, sample range, size, response rate, study design, and findings). The spreadsheet was used by the authors to narratively summarize the findings. Meta-analysis was not conducted due to heterogeneity of outcomes, indicators, and samples.

We assessed the quality of the literature across different studies, and commented on strengths and limitations of the collective evidence. Quality criteria was single-rated and based on combined criteria from two reviews, which particularly suited this review given the variety of indicators, outcomes and analyses included [23, 24]. These criteria were adapted to the research questions in the current review, and the following quality criteria were particularly emphasized as positive: (i) objective measures of employee heath; (ii) objective measures of patient safety; (iii) interventional or longitudinal observational studies; (iv) in case of longitudinal studies, repeated exposure and outcome measures; (v) control variables, when relevant; (vi) large sample size; and (vii) risk of bias within and across studies.

## Results

**Fig. 1:**
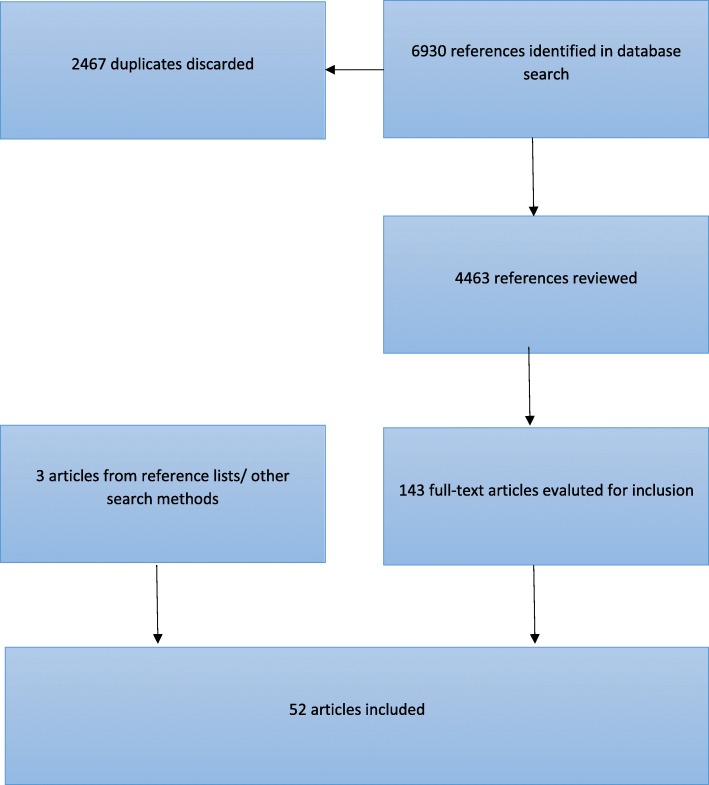
Selection process

The database search resulted first in 6930 studies, and later 4463 references, after discarding duplicates. Two independent researchers screened the studies. On consensus, 143 article titles and abstracts were eligible for full-text screening. Fifty-two articles fulfilled our inclusion criteria. The selection process and the number of articles included at each consecutive step of the systematic literature review are shown in Fig. 1.

Additional file 2 Appendix 2 shows data extraction from included studies and summarize findings. The included studies analyze the relationship between working time, employee health, and patient safety in different ways. None of the studies directly investigated the research question by studying whether employee health mediated the relationship between working time and patient safety. We grouped studies according to the types of indirect investigations of the research question “Is the relationship between working time arrangements and patient safety accounted for by employee health?”: 1) independent investigations of working time arrangements and employee health, and of working time arrangements and patient safety; 2) investigations of working time arrangements on both employee health and patient safety; 3) investigations of working time arrangements and employee health as two explanatory variables for patient safety; 4) combination studies; 5) other studies.

Because each of these approaches imply different perspectives on the correlation between working time, employee health and patient safety, we first present the results separately.

### Independent investigations of working time and employee heath, and of employee health and patient safety

In five studies, the authors analyzed the correlation between working time and employee health, and between employee health and patient safety. All combined, the articles support the idea that some working time arrangements, particularly night work and the total number of working hours per week, are related to negative health consequences like lack of sleep, poor quality of sleep, sleepiness and burnout. The studies also find that the above-mentioned sleep variables are related to an increased risk of error in patient care [25–29]. The articles suggest that working time arrangements may lead to reduced patient safety by adversely affecting employee health, but do not go far in testing the idea.

#### Quality assessment

The sample sizes vary between 180 and just over 3600 employees. Both outcome measures are self-reported in all articles. Patient safety is measured as errors. All five articles are based on cross-sectional data, and none of the five assess whether health mediates the relationship between working time and patient safety. Self-reported outcomes may lead to biased results, for example, if employees who have admitted making an error are more prone to reporting having been sleepy or having worked too long.

### Investigations of working time arrangements on both employee health and patient safety

In all, 21 studies investigated whether working time arrangements were related to both patient safety and employee health. Of the 21 studies, there were six intervention studies and six that used longitudinal methods, while the other nine were cross-sectional studies.

A prospective group-randomized intervention found that intensive care doctors who had weekends off during a 14-day work period reported significantly less burnout and did not show significantly improved patient outcomes in the unit (length of stay and mortality) [30]. The sample was considerably lower (*N* = 45) than what the authors had estimated as being necessary to find significant results.

In another group-randomized controlled study, doctors’ work hours where reduced from 56 to 48 h per week in an intervention group [31]. The doctors in the intervention group slept an average of 30 min more per day. This is a substantial difference but, due to a low sample size (19 doctors), it is not significant. Inspection of over 400 patient journals showed significantly fewer medical errors and negative patient incidents among the intervention group doctors.

In a randomized controlled trial, 47 doctors where divided into shifts of 12, 16 and 24 h [32]. The results showed no significant difference between shift length for adverse incidents, burnout or doctors’ sleepiness, but showed more frequent negative mental symptoms among the doctors who worked 24-h shifts.

In a retrospective cohort study of around 60 doctors, the total number of doctors on sick leave increased by 90% when the working hours were reduced from 56 to 48 h per week [33]. The authors found no change in the patients’ situation (in mortality, length of stay or readmission). A rise in sick leave may be due to the fact that work intensity increases when the working time is reduced – due to lack of increase in staffing.

A Garland, D Roberts and L Graff [34] investigated two types of working time arrangements for 24 intensive care doctors. Either the same doctor worked 24 h a day for seven days in a row, with on-call duties at home (standard), or one doctor worked the day shift for seven days straight while another doctor worked the night shift (shift/intervention). Each team of doctors rotated between the two arrangements. The doctors had significantly lower burnout when they worked shifts, while there was no difference in patient outcomes.

The sixth intervention study stands out from the others. It was a group-randomized intervention of over 4000 doctors and more than 100,000 patients in the USA [35]. The point of the intervention was to compare American working time rules for doctors in training (maximum 80 h per week, 16-h shifts) with rules that were more liberal on shift length and rest between shifts, but which maintained 80 h per week. The study showed no significant difference in patient outcomes in terms of objective measures such as mortality, complications and infections. Neither did itshow any significant difference in general well-being among doctors, nor in terms of doctors’ fatigue adversely affecting patient safety. Noticeably, by keeping the number of hours per week constant while allowing the doctors to work longer days and have shorter rests between shifts, the intervention also allowed for the doctors to work fewer days per week. These results are in line with previous studies of the American working time reform [36].

Collectively, the intervention study results indicate that the working time interventions that lead to improved health (less burnout and more sleep) also frequently have positive consequences for patients [30, 31], with exceptions [34]. Furthermore, working time interventions with little or negative outcomes for employees do not have positive consequences for patient safety either [32, 33, 35]. Consistently small selections (between 19 and 58 persons) and non-significant differences in the first five intervention studies make it difficult to draw conclusions.

Six studies used longitudinal methods. One study testing the same surgeons before and during a 17-h night shift found significant increases in sleepiness during the shift, but no significant change in job performance or number of errors in a simulated test [37]. Another study tested the same anesthesiologists after a night off and after a night shift, finding that the doctors had slept significantly less after the night shift, and performed significantly worse in a simulated test [38].

In one longitudinal study, working time, employee health and patient outcomes were measured monthly among over 2700 doctors for a year [39]. During the months they worked more long shifts (> 24 h), the doctors slept fewer hours, dozed off more often at work and reported more medical errors. Moreover, there was a greater probability that such errors led to negative patient outcomes. Another study tested surgeons before/at the start of a 24-h shift and the morning after a 24-h shift [40]. Surgeons who slept less after a 24-h shift, were more fatigued and made more errors during a simulated test following the shift.

AL Garden, BJ Robinson, LJ Kappus, I Macleod and PH Gander [41] studied 12 doctors who worked three consecutive shifts of 15 h, 9 h and 15 h, respectively. The doctors were measured at the start of the first 15-h shift and at the end of the second 15-h shift. The doctors had slept significantly less at the second measurement, though the difference was small and described by the authors as likely inconsequential. There was a non-significant tendency for the doctors to perform a less adequate check of the equipment after the second 15-h shift and, otherwise, little difference in performance. In all, 502 nurses were followed through a diary study in which they reported on their working time, errors and sleepiness over a 14-day period [42]. When the nurses had worked 12.5 h or more, the odds of making an error, being sleepy and dozing off at work significantly increased.

Of the six longitudinal studies, four showed significant correlation between the working time variables (long shifts and night shifts) and both employee health (sleepiness, sleep, fatigue) and patient safety (self-reported errors and performance in simulated tests). One study only showed a change in employee health, and the last study showed small differences of unclear significance in both employee health and patient safety. The findings from the longitudinal studies indicate that, when working time arrangements are related to negative employee health outcomes, they are also often related to negative patient outcomes.

Of nine cross-sectional studies there were seven that examined employee health in the form of sleep, lack of sleep, sleep quality and burnout [43–49]. Six of seven studies found at least one significant correlation between working time (total hours worked per week, shift length, night shift, rotating shifts), and both employee health and patient safety (errors, errors with negative consequences for patients, wounds, infections). For example, in a selection of 3710 nurses, shifts over 13 h were significantly related to higher burnout levels, low general safety evaluation and increased risk of lost information during handover between shift and infections [48]. Two of these six studies additionally found working time variables that were significantly related to only employee health, or to neither patient safety nor employee health. P Gander, H Purnell, A Garden and A Woodward [49] found that the number of work days in the past two weeks and 14-h shifts were not significantly related to sleepiness or errors. The number of working hours in the past two weeks and shift rotation were significantly related to sleepiness, but not to errors. Having more frequent night shifts (more than 2 nights per week) was related to both sleepiness and errors. Another study [43] found no significant difference in burnout or patient safety across shift types, but did find reduced sleep quality among night-shift personnel.

Three of the cross-sectional studies included employee health measures that were more similar in nature to patient safety and errors in patient care (e.g., nurses mistakenly sticking themselves with needles). Two of three studies showed that having more work hours per week was related to increased risk of both employee injury on the job and errors in patient care [44, 50], while one study showed that having more work hours per week was only significantly related to negative patient outcomes (medication errors, falls and pressure sores) [51]. These three studies had sample sizes between 173 and just over 11,500 participants.

The majority of the studies (12 of 20) that investigated working time as a possible factor in both patient safety and employee health consistently found a significant correlation to both. This primarily refers to working time arrangements such as night shifts, rotating shifts, high number of hours worked per week and long shift length. The working time arrangements are related to adverse health outcomes like lack of sleep, poor sleep quality, sleepiness, fatigue and burnout, as well as errors in patient care and adverse patient consequences such as wounds and infections.

Some studies also found a significant correlation between a working time arrangement and employee health – without finding any correlation to patient safety. However, only two studies found a significant correlation between working time arrangement and patient safety while not finding a significant correlation between working time arrangement and employee health. There is one study in which a very small sample made it difficult to draw conclusions [31], and one study that looked at employee injuries/accidents rather than sleep or burnout [51]. Taken together, the studies suggest that working time arrangements that are detrimental to employee health often lead to negative patient outcomes as well. We seldom see a significant correlation between working time arrangements and negative patient outcomes when working time arrangements are not also related to employee health. Even if the studies did not explicitly test employee health as a mediating factor between working time and patient safety, the results support such a relationship.

#### Quality assessment

In the six intervention studies, patient outcomes are gathered from hospital records, patient files and/or assessed by a third party; however, employee health outcomes were still self-reported in all but one study.

All six of the longitudinal studies used repeated measures of working time, employee health, and patient safety, allowing each employee to serve as his/her own control. When the doctor was his/her own control, each doctor was compared with him−/herself to prevent fixed differences between employees affecting the results. Four of the six longitudinal studies used more objective measures of patient safety (typically, simulator performance review by a blind third party), while two also used objective records of employee health (wristbands to measure sleep). The six studies comprised selections of from 12 to about 2700 persons.

The cross-sectional studies relied solely on self-reported measures. Half of the cross-sectional studies also used few or no control variables.

These combined results are strengthened by a high percentage of longitudinal studies, and the use of objective outcome measures in several studies. The intervention studies gave more mixed results and are more difficult to draw conclusions from. In particular, small samples in several interventions and some of the longitudinal studies complicate the interpretation and generalizing ofnull findings.

### Investigations of working time arrangements and employee health as two explanatory variables for patient safety

In all, eight studies (nine articles) looked at both working time and employee health as explanatory variables for patient safety. Of special interest in these studies is what happens to the correlation between working time and patient safety when employee health is controlled for. If employee health explains the correlation between working time and patient safety, there will no longer be a correlation between working time and patient safety when employee health is controlled for.

C Arakawa, Y Kanoya and C Sato [52] showed that frequent overtime and several night shifts per month were related to more perceived errors. However, the correlation was no longer significant when health and other control variables were included. Multiple health indicators (sick leave, pain, undergoing treatment) were significantly related to medical errors, also after allowing for control variables. The final analysis included a number of control variables (such as age, work experience and staffing) and no odds ratio for an unadjusted analysis was provided. It is therefore difficult to say how great a reduction there was in the correlation between working time and errors, or how much of the reduction could be attributed to employee health.

M Arimura, M Imai, M Okawa, T Fujimura and N Yamada [53] found no association between overtime and frequency of night shifts (for shift workers) and medical errors. Sleepiness and lower quality of sleep were related to increased probability of error in a simple correlation analysis, but not after having controlled for different variables. Particularly noteworthy, reduced mental health and shift work were both significantly related to increased risk of error, also after having controlled for each other and for several control variables such as sleepiness, sleep quality, free time and sleep requirements. There was an independent correlation between shift work (working rotating shifts as compared to day shifts) and increased medical errors, beyond what could be explained by the employees’ general health, sleepiness and sleep quality. The authors did not provide the odds ratio for an unadjusted analysis; thus, it is more difficult to say whether there was a reduction in the correlation between working time and errors after inclusion of control variables.

LD Scott, C Arslanian-Engoren and MC Engoren [54] found that sleepiness during the day, lack of sleep and 12-h shifts were each associated with higher odds that a nurse regretted a clinical decision made while sleepy. When all the variables were included in the same model, such that they controlled for each other, it was only the 12-h shifts that were associated with regretting a decision. The authors concluded that 12-h shifts could lead to sleepiness, but that it was not the sleepiness that led to the incidents the nurses regretted. In contrast, night shifts were only associated with regret in a single correlation analysis, and not after sleepiness and other control variables were added to the model.

R Jagsi, BT Kitch, DF Weinstein, EG Campbell, M Hutter and JS Weissman [55] found that fatigue and the type of shift rotation were both significantly related to increased risk of near-errors, also after having controlled for each other and other control variables. Type of rotation and working more than 80 h a week were related to several negative patient incidents, before and after inclusion of control variables. Fatigue was only related to negative patient incidents in a simple correlation analysis, and not after having controlled for working time and other control variables.

In an analysis that controlled for shift length, MZ Ramadan and KS Al-Saleh [56] found that lack of sleep was significantly related to the frequency of errors. In another analysis in the same article, the authors found that the number of work hours per week, but not the number of hours slept, was significantly related to frequency of errors. The analysis was not shown in its entirety and the control variables were not clearly presented.

K Suzuki, T Ohida, Y Kaneita, E Yokoyama, T Miyake, S Harano, Y Yagi, E Ibuka, A Kaneko, T Tsutsui, et al. [57] found that the risk of work accidents (incorrect medication or use of medical equipment, incorrect identification of patients, needle stick accidents) was significantly higher for those with poor mental health and for those who worked shifts, also after controlling for each other and other control variables (including several sleep variables, age and civil status). In the adjusted analysis (i.e. with control variables), the significance of shift work was reduced by 30% (OR from 2,54 to 1,78), while the significance of mental health was reduced by 10% (OR from 1,72 to 1,55). Using the same data, K Suzuki, T Ohida, Y Kaneita, E Yokoyama and M Uchiyama [58] found an independent correlation between medication errors and both shift work and extreme sleepiness. The strength of the correlation between shift work and errors was reduced by 16% (OR from 2.18 to 1.78) in the adjusted model, but there was no reduction for sleepiness. Shift work was no longer significantly related to incorrect use of medical equipment when sleepiness and other control variables were included in the analysis.

J Wen, Y Cheng, X Hu, P Yuan, T Hao and Y Shi [59] found that the risk of error increased when employees worked 60 h or more per week or had considerable burnout. Both relationships remained significant after controlling for each other. When the authors controlled for gender, hospital type and burnout, the odds ratio decreased by 28% (from 2.29 to 1.65) for the correlation between working time and errors. Even though the correlation between working time and errors was still significant, the control variables may explain a considerable part of that. For burnout, the reduction in the same analysis was 18% (from 2.79 to 2.28).

In one study, the correlation between working time variables (overtime and night shifts) and patient safety was no longer significant when health (sick leave, receiving treatment, bodily pain) was controlled for [52]. In the other five studies where control variables were clearly presented, an independent correlation was found between working time variables (shift work, 12-h shifts, 60 h per week and type of rotation) and patient safety, even after health (mental health, burnout, sleepiness, sleep quality, lack of sleep and fatigue) was controlled for [53–59, 57–59]. In the studies that provided odds ratios for both the unadjusted and adjusted analyses, there was a considerable reduction in the strength of the correlation between working time variables and patient safety when the control variables (including health) were included. None of the studies addressed how great a proportion may be explained by health.

Collectively, the studies therefore indicate that while health variables may explain parts of the correlation between working time arrangements and patient safety, they do not seem to explain the entire correlation.

#### Quality assessment

All of the studies were based on cross-sectional data and self-reporting. In four of the papers the control variables are not clearly specified. Sample sizes varied between 138 and 6445.

### Combination studies

We identified seven articles that combined several analyses discussed above. These articles sprung out ofsix studies that investigated a number of possible correlations between working time, employee health and patient safety in the same study.

J Dorrian, N Lamond, C van den Heuvel, J Pincombe, AE Rogers and D Dawson [60] found no significant correlation between shift length and amount of sleep. Additionally, they found no significant correlation between type of shift and shift length, on the one hand, and errors on the other. Amount of sleep was significantly adversely related to the number of reported errors. J Dorrian, C Tolley, N Lamond, C van den Heuvel, J Pincombe, AE Rogers and D Drew [61] also found, in part of the same data material, that shift length was significantly related to struggling to stay awake and that struggling to stay awake was significantly related to errors. J Dorrian, C Tolley, N Lamond, C van den Heuvel, J Pincombe, AE Rogers and D Drew [61] did not find any direct correlation between shift length and errors either.

DM Houston and SK Allt [62] found that weekly working time was not associated with health or cognitive error. Cognitive errors were related to medical errors, but general health status did not have an independent correlation to medical errors when cognitive error was controlled for.

DA Kalmbach, JT Arnedt, PX Song, C Guille and S Sen [63] found that weekly working time (> 70 h) was significantly related to less sleep and depression. Weekly working time, length of sleep, and depression were all significantly related to errors in patient care in an analysis without control variables.

LI Keshk and DS Abd El-Moneem [64] showed a significant correlation between weekly working time and physical fatigue, but not concentration fatigue. Concentration fatigue, and not physical fatigue, was significantly related to medication errors. Weekly working time was not significantly related to medication errors either.

Y Kaneita and T Ohida [65] showed that increased number of hours worked per day was related to less sleep, greater lack of rest due to lack of sleep, and more insomnia. The number of days with on-call duty was also related to less sleep and greater lack of rest due to lack of sleep. Furthermore, lack of rest due to lack of sleep and insomnia was related to medical errors. In the final logistic regression, working 10 h or more per day (compared with six to eight hours), and having on-call duty two to seven times per month (compared to never) were also significantly related to medical error. The correlation held after having controlled for insomnia, lack of rest due to lack of sleep, length of sleep and other control variables such as age and gender as well. The unadjusted OR was not provided in the article. It may be interpreted that working time has an indirect correlation with medical errors (via sleep) as well as a direct correlation that cannot be explained by these variables.

AL Weaver, SE Stutzman, C Supnet and DM Olson [66] found no difference between night-shift workers and day-shift workers in the number of hours slept, sleep quality or self-reported errors. Sleep quality, but not the number of hours slept, was significantly related to self-reported errors.

Combined, these six studies paint a similar picture to what we find in the studies reviewed above. When the working time arrangement is not related to reduced health, it is seldom related to reduced patient safety. If the working time arrangement is related to reduced health, it is often related to reduced patient safety as well. The results further support that the type of health is important: there were no correlation between working time and errors when working time was only related to physical fatigue – which was not related to patient safety [64]. Again, we still see a correlation between working time and patient safety that is also present after health (length of sleep, lack of sleep and insomnia) is controlled for.

#### Quality assessment

Three of the articles were prospective cohort studies, two were from the same diary study, and the rest were cross-sectional. The sample size ranged from 20 to 3286. All but one study used self-reported measures of employee health (one used wristbands), and all studies but one used self-reported measures of patient safety (one used third-party observations).

### Other studies

Eleven studies did not fit into the other category groups, but nonetheless informed the research question [67–77].

Two qualitative studies [73, 74] closely investigate how harmful working time arrangements can have a negative impact on patient safety. In one study, nurses in Canada reported on how they had perceived the physical consequences of overtime; particularly physical pain such as neck and shoulder pain, fatigue, hunger, dehydration and increased susceptibility to illness [73]. When the overtime led to less sleep and more rushing around, they got more fatigued and were less capable of doing the job correctly. For example, when tired they were less inclined to follow procedures for such things as preventing infections, their skills were reduced and there was an increased probability of making errors. The nurses also reported other negative consequences for patient safety such as poorer communication, and a more task-oriented rather than patient-oriented focus. In another study surgeons talked about fatigue after night shifts, and how the fatigue adversely affected their cognitive abilities; in particular, many experienced difficulties concentrating and reduced communication skills [74].

Three studies outlined an alternative explanation for the correlation between working time, employee health and patient safety. The studies highlighted how low levels of patient safety could also adversely affect employee health. Two studies showed that doctors who had been sued for maltreatment during the past 24 months, or who had some experience with incorrect treatment, had a higher risk of burnout [75, 76]. The third study documented doctors’ feelings in the aftermath of their most memorable mistake [77]. The majority experienced feelings such as shame, disappointment, irritation and guilt. However, 14% reported that such episodes had affected their sleep, while 18% reported effects on their mood. In more than 80% of the cases, the error had not led to patient injury.

The last three studies are cross-sectional, based on self-reporting. They do not test causal directions, but are nonetheless interesting because they present an alternative explanation for the correlation between working time, employee health and patient safety.

## Discussion

The purpose of this systematic literature review was to investigate whether the hypothesis that employee health accounts for the relationship between working time arrangements and patient safety is empirically supported. The results from the 52 articles that met the inclusion criteria show several working time arrangements that are related to negative employee health outcomes. A number of the adverse employee health outcomes were also related to reduced patient safety, particularly an increased number of errors made by health personnel.

Additionally, qualitative studies exemplify how employee health can impact patient care, when fatigue reduces health workers’ cognitive skills (e.g. reduced alertness and concentration) and changes behavior (e.g., being less inclined to follow safety procedures when tired, reducing focus and communication) [73, 74]. These results suggest a possibility that working time arrangements may lead to negative consequences for the patients by reducing employee health.

### Is there support for the hypothesis that the relationship between working time and patient safety is explained by employee health?

We found no studies that directly tested whether employee health mediates the correlation between working time arrangements and patient safety. A number of studies nonetheless provide information on the relationship between the consequences of working time arrangements for employee health and the consequences of working time arrangements for patient safety, without using a mediation model.

The results show that working time arrangements related to employee health are often also related to patient safety, while working time arrangements not related to employee health rarely are related to patient safety. If some working time arrangements are negative for patient safety *because* they are negative for employee health, we should expect to find that working time arrangements are only adversely related to patient safety when they also are adversely related to employee health. This was supported by the results. In most of the studies that tested whether specific working time arrangements were related to employee health *and* patient safety, we found a correlation with both outcome measures. However, while a few studies also found a correlation only between a working time arrangement and employee health, studies seldom found a correlation between a working time arrangement and patient safety when the working time arrangement was not adversely related to employee health. This supports the assumption that working time arrangements may lead to reduced patient safety because they adversely impact employee health. However, it also underscores that negative consequences for employee health do not necessitate negative patient outcomes.

Results showed a significant correlation between working time arrangements and patient safety after having controlled for employee health. If some working time arrangements are negative for patient safety only because they are negative for employee health, the correlation between working time arrangements and patient safety should no longer be significant when employee health is controlled for. Indeed, we did not find support for this. This may be explained by several health factors having an independent effect on patient safety and studies not controlling for all aspects of employee health, however several studies did include multiple relevant health measures (e.g. general health, sleepiness and sleep quality in the same analyses) [78–82]. The results suggest that several working time arrangements have a direct independent correlation on patient safety beyond what can be explained by employee health. As discussed in the introduction, one possible explanation may be that other factors, such as work environment, are affected by working time arrangements [43] and that work environment, in turn, affects both employee health [83, 84] and patient safety [85, 86]. For example, reduced professional support could potentially have an adverse effect on the health of employees and the professional decisions they make.

It is nonetheless important to note that a few studies showed both unadjusted (without control variables) and adjusted (with control variables) odds ratios for the correlation between working time and patient safety. They found that the strength of the correlation between the working time arrangement and patient safety was considerably reduced when the control variables were added. This suggests that part of the correlation between working time and patient safety may be explained by the control variables. However, because the analysis often included several control variables, it is difficult to say how great a portion of the relationship may be explained by employee health.

### Alternative causal correlations

The results in this literature review suggest that some working time arrangements may lead to adverse consequences for patients, in part by reducing employee health. However, this is just one explanation for the relationship between the three variables. Even in longitudinal studies, employee health and patient safety are generally measured at the same timepoint, which makes it methodologically difficult to distinguish between what comes first and last, thereby confusing potential causal correlations. Consequently, potential causal correlations must be discussed theoretically. The most common hypothesis provided in the identified papers is that working time leads to reduced health, and reduced health leads to reduced patient safety. However, other potential explanations are also suggested. A possible alternative explanation is that health affects working time (in that those with poorer health work shorter weeks, avoid night shifts, etc.). Such a selection effect may have been present when H Admi, O Tzischinsky, R Epstein, P Herer and P Lavie [68] found more health issues among daytime workers (compared to shift workers), a correlation that was no longer significant when the authors controlled for age, among other things.

We can also expect that patient safety affects employee health if low levels of patient safety and the experience of having made medical errors takes a toll on employees. Such a causal correlation is supported, in part, by D McCawley, AM Cyna, S Prineas and S Tan [77], who documented the negative feelings employees experience following their most memorable error. It is also supported by CM Balch, MR Oreskovich, LN Dyrbye, JM Colaiano, DV Satele, JA Sloan and TD Shanafelt [75], who showed that doctors who had been sued in the past 24 months had a higher risk of burnout, symptoms of depression and suicidal thoughts. Again, we can also speculate whether working time arrangements may have an independent effect on both employee health and patient safety, for example, due to differences in work environment and staffing.

### Limitations

Some limitations of the current review are worth highlighting. With respect to the quality of evidence and risk of bias, the most central challenge is the lack of studies with the purpose of testing whether employee health mediates the relationship between working time and patient safety. The study designs applied in the papers we have identified are, therefore, not ideal for our research questions (i.e., employee health and patient safety are generally measured at the same time, even in longitudinal studies and randomized controlled trials).

The quality of evidence also varies between the different findings in our review. We found that working time arrangements related to employee health are frequently also related to patient safety; however, working time arrangements that are unrelated to employee health rarely are related to patient safety. This finding is supported by several longitudinal studies utilizing fixed effect methods and outcome measures with less risk of bias than self-report (i.e. simulator performance review by a blind third party). We also found that there is an independent relationship between working time arrangements and patient safety after employee health is controlled for. This finding is supported only by cross-sectional studies using self-report measures.

While a central aspect of the review is patient safety, relatively few articles actually measured patient outcomes. Most of the studies measured employees’ self-reported errors and near errors. This may have had significance for the results if there were imbalances in terms of who reported such errors, or which errors employees noticed and reported on. Particularly in the cases where employees are specifically asked to provide fatigue-related errors, it is natural that fatigued employees are more likely to report such errors. We can also speculate as to whether employees more easily discover errors that happen during their shifts, rather than in the transition between shifts, and that, as such, errors connected to frequency of shift change between caretakers may be underestimated.

Additionally, we acknowledge that working time arrangements may influence other employee and patient outcomes than those investigated in this review, such as patient satisfaction, general quality of the patient care, empathy, kindness and patience towards patients [69, 87].

## Conclusion

Our findings support that adverse working time arrangements for employee health, particularly those linked to insomnia, fatigue and burnout, are generally associated with reduced patient safety as well. The findings suggest that working time arrangements may lead to reduced patient safety, partly because they adversely impact employee health. However, no studies directly tested a mediation model. Moreover, several studies clearly supported that employee health alone cannot explain the relationship between working time arrangements and patient safety. Further research should directly examine employee health as a mediating variable between working time arrangements and patient safety, as well as other potential explanations. Shiftwork is unavoidable within the health sector – a better understanding of why some working time arrangements seem to reduce patient safety is a crucial first step to reduce harmful effects on the long run.

## Additional files

Additional file 1: Appendix 1. Search history. Keywords, databases and number of results. (DOCX 53 kb)

Additional file 2: Appendix 2. Table of studies. Table of data extracted from included studies. (DOCX 160 kb)

## References

[1] Bae SH, Fabry D (2014). Assessing the relationships between nurse work hours/overtime and nurse and patient outcomes: systematic literature review. Nurs Outlook.

[2] de Cordova PB, Phibbs CS, Bartel AP, Stone PW (2012). Twenty-four/seven: a mixed-method systematic review of the off-shift literature. J Adv Nurs.

[3] Lie J-AS, Arneberg L, Goffeng LO, Graveseth HM, Lie A, Ljoså GH, Matre D: Arbeidstid og helse. Oppdatering av en systematisk litteraturstudie. In*.*, vol. 15. Oslo: Statens arbeidsmiljøinsitutt; 2014.

[4] Lie J-AS, Gulliksen E, Bast-Pettersen R, Skogstad M, Tynes T, Wagstaff AS (2008). Arbeidstid og helse. En systematisk litteraturstudie. In*.*, vol. 9.

[5] Solovieva S, Lallukka T, Virtanen M, Viikari-Juntura E (2013). Psychosocial factors at work, long work hours, and obesity: a systematic review. Scand J Work Environ Health.

[6] Clendon J, Gibbons V (2015). 12 h shifts and rates of error among nurses: a systematic review. Int J Nurs Stud.

[7] Lee A, Myung SK, Cho JJ, Jung YJ, Yoon JL, Kim MY (2017). Night shift work and risk of depression: meta-analysis of observational studies. J Korean Med Sci.

[8] Angerer P, Schmook R, Elfantel I, Li J (2017). Night work and the risk of depression. Dtsch.

[9] Vyas MV, Garg AX, Iansavichus AV, Costella J, Donner A, Laugsand LE, Janszky I, Mrkobrada M, Parraga G, Hackam DG (2012). Shift work and vascular events: systematic review and meta-analysis. BMJ.

[10] de Cordova PB, Bradford MA, Stone PW (2016). Increased errors and decreased performance at night: a systematic review of the evidence concerning shift work and quality. Work.

[11] Wickens CD, Hutchins SD, Laux L, Sebok A (2015). The impact of sleep disruption on complex cognitive tasks: a meta-analysis. Hum Factors.

[12] Cho E, Lee H, Choi M, Park SH, Yoo IY, Aiken LH (2013). Factors associated with needlestick and sharp injuries among hospital nurses: a cross-sectional questionnaire survey. Int J Nurs Stud.

[13] Vahey DC, Aiken LH, Sloane DM, Clarke SP, Vargas D (2004). Nurse burnout and patient satisfaction. Med Care.

[14] Barker LM, Nussbaum MA (2011). Fatigue, performance and the work environment: a survey of registered nurses. J Adv Nurs.

[15] Ahmed N, Devitt KS, Keshet I, Spicer J, Imrie K, Feldman L, Cools-Lartigue J, Kayssi A, Lipsman N, Elmi M (2014). A systematic review of the effects of resident duty hour restrictions in surgery: impact on resident wellness, training, and patient outcomes. Ann Surg.

[16] Harris R, Sims S, Parr J, Davies N (2015). Impact of 12h shift patterns in nursing: a scoping review. Int J Nurs Stud.

[17] Hamilton P, Eschiti VS, Hernandez K, Neill D (2007). Differences between weekend and weekday nurse work environments and patient outcomes: a focus group approach to model testing. The Journal of Perinatal & Neonatal Nursing.

[18] Aiken LH, Cimiotti JP, Sloane DM, Smith HL, Flynn L, Neff DF (2011). Effects of nurse staffing and nurse education on patient deaths in hospitals with different nurse work environments. Med Care.

[19] Conti G, Heckman J, Urzua S (2010). The education-health gradient. Am Econ Rev.

[20] Lange R (2012). Health-related quality of life within the first five years following polytrauma and mild TBI in US military service members. Arch Phys Med Rehabil.

[21] Bolster L, Rourke L (2015). The effect of restricting Residents' duty hours on patient safety, resident well-being, and resident education: an updated systematic review. J Grad Med Educ.

[22] Harris J, Staheli G, LeClere L, Andersone D, McCormick F: What effects have resident work-hour changes had on education, quality of life, and safety? A systematic review (Provisional abstract). In: Database of Abstracts of Reviews of Effects. 2014: epub. 10.1007/s11999-014-3968-0 PMC4385350 25269530

[23] Ariens GA, Van Mechelen W, Bongers PM, Bouter LM, Van Der Wal G (2000). Physical risk factors for neck pain. Scand J Work Environ Health.

[24] Dewa CS, Loong D, Bonato S, Hees H. Incidence rates of sickness absence related to mental disorders: A systematic literature review. BMC Public Health. 2014;14(1). 10.1186/1471-2458-14-205 PMC3939632 24571641

[25] Asaoka S, Aritake S, Komada Y, Ozaki A, Odagiri Y, Inoue S, Shimomitsu T, Inoue Y (2013). Factors associated with shift work disorder in nurses working with rapid-rotation schedules in Japan: the nurses' sleep health project. Chronobiol Int.

[26] Baldwin DC, Daugherty SR (2004). Sleep deprivation and fatigue in residency training: results of a national survey of first- and second-year residents. Sleep.

[27] Chen I, Vorona R, Chiu R, Ware JC (2008). A survey of subjective sleepiness and consequences in attending physicians. Behav Sleep Med.

[28] de Oliveira GS, Jr., Chang R, Fitzgerald PC, Almeida MD, Castro-Alves LS, Ahmad S, McCarthy RJ: The prevalence of burnout and depression and their association with adherence to safety and practice standards: a survey of United States anesthesiology trainees. Anesth Analg 2013, 117(1):182–193. 10.1213/ANE.0b013e3182917da9 23687232

[29] Qureshi HA, Rawlani R, Mioton LM, Dumanian GA, Kim JY, Rawlani V (2015). Burnout phenomenon in U.S. plastic surgeons: risk factors and impact on quality of life. Plast Reconstr Surg.

[30] Ali NA, Hammersley J, Hoffmann SP, O'Brien JM, Phillips GS, Rashkin M, Warren E, Garland A (2011). Midwest critical care C: continuity of care in intensive care units: a cluster-randomized trial of intensivist staffing. Am J Respir Crit Care Med.

[31] Cappuccio FP, Bakewell A, Taggart FM, Ward G, Ji C, Sullivan JP, Edmunds M, Pounder R, Landrigan CP, Lockley SW (2009). Implementing a 48 h EWTD-compliant Rota for junior doctors in the UK does not compromise patients' safety: assessor-blind pilot comparison. Qjm.

[32] Parshuram CS, Amaral AC, Ferguson ND, Baker GR, Etchells EE, Flintoft V, Granton J, Lingard L, Kirpalani H, Mehta S (2015). Patient safety, resident well-being and continuity of care with different resident duty schedules in the intensive care unit: a randomized trial. Cmaj.

[33] McIntyre HF, Winfield S, Te HS, Crook D (2010). Implementation of the European working time directive in an NHS trust: impact on patient care and junior doctor welfare. Clin Med.

[34] Garland A, Roberts D, Graff L (2012). Twenty-four-hour intensivist presence: a pilot study of effects on intensive care unit patients, families, doctors, and nurses. Am J Respir Crit Care Med.

[35] Bilimoria KY, Chung JW, Hedges LV, Dahlke AR, Love R, Cohen ME, Hoyt DB, Yang AD, Tarpley JL, Mellinger JD (2016). National Cluster-Randomized Trial of duty-hour flexibility in surgical training. N Engl J Med.

[36] Ahmed N, Devitt KS, Keshet I, Spicer J, Imrie K, Feldman L, Cools-Lartigue J, Kayssi A, Lipsman N, Elmi M (2014). A systematic review of the effects of resident duty hour restrictions in surgery: impact on resident wellness, training, and patient outcomes. Ann Surg.

[37] Amirian I, Andersen LT, Rosenberg J, Gögenur I (2014). Laparoscopic skills and cognitive function are not affected in surgeons during a night shift. J Surg Educ.

[38] Arzalier-Daret S, Buleon C, Bocca ML, Denise P, Gerard JL, Hanouz JL (2017). Effect of sleep deprivation after a night shift duty on simulated crisis management by residents in anaesthesia A randomised crossover study. Anaesth Crit Care Pain Med.

[39] Barger LK, Ayas NT, Cade BE, Cronin JW, Rosner B, Speizer FE, Czeisler CA (2006). Impact of extended-duration shifts on medical errors, adverse events, and attentional failures. PLoS Med.

[40] Eastridge BJ, Hamilton EC, O'Keefe GE, Rege RV, Valentine RJ, Jones DJ, Tesfay S, Thal ER (2003). Effect of sleep deprivation on the performance of simulated laparoscopic surgical skill. Am J Surg.

[41] Garden AL, Robinson BJ, Kappus LJ, Macleod I, Gander PH (2012). Fifteen-hour day shifts have little effect on the performance of taskwork by anaesthesia trainees during uncomplicated clinical simulation. Anaesth Intensive Care.

[42] Scott LD, Rogers AE, Hwang WT, Zhang Y (2006). Effects of critical care nurses' work hours on vigilance and patients' safety. Am J Crit Care.

[43] Gomez-Garcia T, Ruzafa-Martinez M, Fuentelsaz-Gallego C, Madrid JA, Rol MA, Martinez-Madrid MJ, Moreno-Casbas T (2016). Syce, group R: Nurses' sleep quality, work environment and quality of care in the Spanish National Health System: observational study among different shifts. BMJ Open.

[44] Baldwin DC, Daugherty SR, Tsai R, Scotti MJ (2003). A national survey of residents' self-reported work hours: thinking beyond specialty. Acad Med.

[45] Estryn-Behar M, Van der Heijden B, Grp NS: Effects of extended work shifts on employee fatigue, health, satisfaction, work/family balance, and patient safety. Work-a Journal of Prevention Assessment & Rehabilitation 2012, 41:4283–4290. 10.3233/WOR-2012-0724-4283 22317378

[46] Gold DR, Rogacz S, Bock N, Tosteson TD, Baum TM, Speizer FE, Czeisler CA (1992). Rotating shift work, sleep, and accidents related to sleepiness in hospital nurses. Am J Public Health.

[47] Kunaviktikul W, Wichaikhum O, Nantsupawat A, Nantsupawat R, Chontawan R, Klunklin A, Roongruangsri S, Nantachaipan P, Supamanee T, Chitpakdee B et al: Nurses' extended work hours: patient, nurse and organizational outcomes. Int Nurs Rev 2015, 62(3):386–393. 10.1111/inr.12195 25997841

[48] Stimpfel AW, Lake ET, Barton S, Gorman KC, Aiken LH (2013). How differing shift lengths relate to quality outcomes in pediatrics. J Nurs Adm.

[49] Gander P, Purnell H, Garden A, Woodward A (2007). Work patterns and fatigue-related risk among junior doctors. Occup Environ Med.

[50] Olds DM, Clarke SP (2010). The effect of work hours on adverse events and errors in health care. J Saf Res.

[51] Bae SH: Presence of nurse mandatory overtime regulations and nurse and patient outcomes. Nurs Econ 2013, 31(2):59–68, 89; quiz 69. 23691746

[52] Arakawa C, Kanoya Y, Sato C: Factors contributing to medical errors and incidents among hospital nurses -Nurses' health, quality of life, and workplace predict medical errors and incidents. Ind Health 2011, 49(3):381–388. 10.2486/indhealth.ms968 21372434

[53] Arimura M, Imai M, Okawa M, Fujimura T, Yamada N (2010). Sleep, mental health status, and medical errors among hospital nurses in Japan. Ind Health.

[54] Scott LD, Arslanian-Engoren C, Engoren MC (2014). Association of sleep and fatigue with decision regret among critical care nurses. Am J Crit Care.

[55] Jagsi R, Kitch BT, Weinstein DF, Campbell EG, Hutter M, Weissman JS (2005). Residents report on adverse events and their causes. Arch Intern Med.

[56] Ramadan MZ, Al-Saleh KS (2014). The association of sleep deprivation on the occurrence of errors by nurses who work the night shift. Curr.

[57] Suzuki K, Ohida T, Kaneita Y, Yokoyama E, Miyake T, Harano S, Yagi Y, Ibuka E, Kaneko A, Tsutsui T (2004). Mental health status, shift work, and occupational accidents among hospital nurses in Japan. J Occup Health.

[58] Suzuki K, Ohida T, Kaneita Y, Yokoyama E, Uchiyama M (2005). Daytime sleepiness, sleep habits and occupational accidents among hospital nurses. J Adv Nurs.

[59] Wen J, Cheng Y, Hu X, Yuan P, Hao T, Shi Y (2016). Workload, burnout, and medical mistakes among physicians in China: a cross-sectional study. Biosci.

[60] Dorrian J, Lamond N, van den Heuvel C, Pincombe J, Rogers AE, Dawson D: A pilot study of the safety implications of Australian nurses' sleep and work hours. Chronobiol Int 2006, 23(6):1149–1163. 10.1080/07420520601059615 17190702

[61] Dorrian J, Tolley C, Lamond N, van den Heuvel C, Pincombe J, Rogers AE, Drew D (2008). Sleep and errors in a group of Australian hospital nurses at work and during the commute. Appl Ergon.

[62] Houston DM, Allt SK: Psychological distress and error making among junior house officers. Br J Health Psychol 1997, 2(Part 2):141–151.

[63] Kalmbach DA, Arnedt JT, Song PX, Guille C, Sen S (2017). Sleep disturbance and short sleep as risk factors for depression and perceived medical errors in first-year residents. Sleep.

[64] Keshk LI, Abd El-Moneem DS: Effect of Nurses' work hours and fatigue on occurrence of medication errors in ICU and medical oncology unit -Cairo University. Life Science Journal-Acta Zhengzhou University Overseas Edition 2012, 9(3):347–355.

[65] Kaneita Y, Ohida T (2011). Association of current work and sleep situations with excessive daytime sleepiness and medical incidents among Japanese physicians. J Clin Sleep Med.

[66] Weaver AL, Stutzman SE, Supnet C, Olson DM (2016). Sleep quality, but not quantity, is associated with self-perceived minor error rates among emergency department nurses. Int Emerg Nurs.

[67] Dean GE, Scott LD, Rogers AE (2006). Infants at risk: when nurse fatigue jeopardizes quality care. Adv Neonat Care.

[68] Admi H, Tzischinsky O, Epstein R, Herer P, Lavie P: Shift work in nursing: is it really a risk factor for nurses' health and patients' safety? Nurs Econ 2008, 26(4):250–257. 18777974

[69] Cammu H, Haentjens P (2012). Perceptions of fatigue - and perceived consequences - among Flemish obstetricians-gynaecologists: a survey. Eur J Contracept Reprod Health Care.

[70] Domen R, Connelly CD, Spence D (2015). Call-shift fatigue and use of countermeasures and avoidance strategies by certified registered nurse anesthetists: a national survey. Aana J.

[71] Seki Y, Yamazaki Y (2006). Effects of working conditions on intravenous medication errors in a Japanese hospital. J Nurs Manag.

[72] Gander PH, Merry A, Millar MM, Weller J (2000). Hours of work and fatigue-related error: a survey of New Zealand anaesthetists. Anaesth Intensive Care.

[73] Lobo VM, Ploeg J, Fisher A, Peachey G, Akhtar-Danesh N (2017). Critical care nurses' perceptions of the outcomes of working overtime in Canada. Nurs Outlook.

[74] Amirian I, A. K. Danielsen, Rosenberg J: Perception of Fatigue Among Surgeons During Night Shifts Ann R Coll Surg Engl 2013, 95(7):(Suppl).

[75] Balch CM, Oreskovich MR, Dyrbye LN, Colaiano JM, Satele DV, Sloan JA, Shanafelt TD (2011). Personal consequences of malpractice lawsuits on American surgeons. J Am Coll Surg.

[76] Chen KY, Yang CM, Lien CH, Chiou HY, Lin MR, Chang HR, Chiu WT: Burnout, job satisfaction, and medical malpractice among physicians. Int J Med Sci 2013, 10(11):1471–1478. 10.7150/ijms.6743 PMC3775103 24046520

[77] McCawley D, Cyna AM, Prineas S, Tan S: A survey of the sequelae of memorable anaesthetic drug errors from the anaesthetist's perspective. Anaesth Intensive Care 2017, 45(5):624–630. 10.1177/0310057X1704500514 28911293

[78] McClelland LE, Switzer FS, Pilcher JJ: Changes in nurses' decision making during a 12-h day shift. Occupational Medicine-Oxford 2013, 63(1):60–65. 10.1093/occmed/kqs189 23117169

[79] Litwiller B, Snyder LA, Taylor WD, Steele LM (2017). The relationship between sleep and work: a meta-analysis. J Appl Psychol.

[80] Nea FM, Kearney J, Livingstone MB, Pourshahidi LK, Corish CA (2015). Dietary and lifestyle habits and the associated health risks in shift workers. Nutr Res Rev.

[81] Lin X, Chen W, Wei F, Ying M, Wei W, Xie X (2015). Night-shift work increases morbidity of breast cancer and all-cause mortality: a meta-analysis of 16 prospective cohort studies. Sleep Med.

[82] Wang F, Zhang L, Zhang Y, Zhang B, He Y, Xie S, Li M, Miao X, Chan EY, Tang JL (2014). Meta-analysis on night shift work and risk of metabolic syndrome. Obes Rev.

[83] Stansfeld S, Candy B. Psychosocial work environment and mental health—a meta-analytic review. Scandinavian Journal of Work, Environment & Health. 2006;(6):443–62. 10.5271/sjweh.1050 17173201

[84] Theorell T, Hammarström A, Aronsson G, Träskman Bendz L, Grape T, Hogstedt C, Marteinsdottir I, Skoog I, Hall C (2015). A systematic review including meta-analysis of work environment and depressive symptoms. BMC Public Health.

[85] McHugh MD, Ma C (2013). Hospital nursing and 30-day readmissions among Medicare patients with heart failure, acute myocardial infarction, and pneumonia. Med Care.

[86] Zúñiga F, Ausserhofer D, Hamers JPH, Engberg S, Simon M, Schwendimann R (2015). The relationship of staffing and work environment with implicit rationing of nursing care in Swiss nursing homes – a cross-sectional study. Int J Nurs Stud.

[87] Stimpfel AW, Sloane DM, Aiken LH (2012). The longer the shifts for hospital nurses, the higher the levels of burnout and patient dissatisfaction. Health Aff (Millwood).

