# Supplementary material for: Healthy working time arrangements for healthcare personnel and patients: a systematic literature review
Source: BMC Health Serv Res. 2019 Mar 27;19:193. doi: 10.1186/s12913-019-3993-5 (PMC6437911; doi:10.1186/s12913-019-3993-5)
Supplement: Supplementary file 1 — Appendix 1. Search history. Keywords, databases and number of results. (DOCX 53 kb) [file 12913_2019_3993_MOESM1_ESM.docx]

# Appendix 1: Data search history

**Databases:** Database(s): Ovid MEDLINE(R) Epub Ahead of Print, In-Process & Other Non-Indexed Citations, Ovid MEDLINE(R) Daily and Ovid MEDLINE(R) 1946 to Present

**Date:** 26 January 2018

**Number of results:** 2896

| # | Searches | Results |
| --- | --- | --- |
| 1 | Work Schedule Tolerance/ | 6261 |
| 2 | shiftwork*.tw,kw,kf. | 708 |
| 3 | nightwork*.tw,kw,kf. | 47 |
| 4 | nightshift*.tw,kw,kf. | 108 |
| 5 | (night* adj2 schedule*).tw,kw,kf. | 199 |
| 6 | (overtime adj3 (hour* or work*)).tw,kw,kf. | 540 |
| 7 | (flextime or flex time or flexitime or flexi time).tw,kw,kf. | 77 |
| 8 | (long* adj2 work* adj2 (hour* or week* or day* or period*)).tw,kw,kf. | 1371 |
| 9 | (short* adj2 work* adj2 (hour* or week* or day* or period*)).tw,kw,kf. | 212 |
| 10 | ((long* or short*) adj2 (workweek* or workday*)).tw,kw,kf. | 36 |
| 11 | (flex* adj1 work*).tw,kw,kf. | 770 |
| 12 | ((extend* or reduc*) adj3 (duty or duties or work*) adj3 (time or hour* or period* or week* or shift* or day* or schedule*)).tw,kw,kf. | 1505 |
| 13 | ((extend* or reduc* or irregular*) adj2 (workweek* or workday*)).tw,kw,kf. | 54 |
| 14 | (irregular* adj3 (work* or shift or shifts)).tw,kw,kf. | 396 |
| 15 | ((unsocia* or antisocia* or anti socia*) adj2 (work* or shift or shifts)).tw,kw,kf. | 41 |
| 16 | ((nonstandard or non standard) adj2 (work* or shift or shifts)).tw,kw,kf. | 103 |
| 17 | ((shift or shifts) adj2 work*).tw,kw,kf. | 6124 |
| 18 | ((shift or shifts) adj2 (rota* or system or systems or schedule* or roster* or fixed or permanent)).tw,kw,kf. | 2784 |
| 19 | ((shift or shifts) adj2 (extend* or pattern* or cycle* or duration* or recover*)).tw,kw,kf. | 1750 |
| 20 | ((shift or shifts) adj2 (evening or night* or late or early or weekend or twilight*)).tw,kw,kf. | 3279 |
| 21 | ((shift or shifts) adj2 (continental or continuous or turnaround or turn around or split)).tw,kw,kf. | 271 |
| 22 | ((shift or shifts) adj length*).tw,kw,kf. | 145 |
| 23 | (hour* adj (shift or shifts or duty or day or days or work* or week*)).tw,kw,kf. | 6198 |
| 24 | (workhour* or worktime).tw,kw,kf. | 133 |
| 25 | (work* adj (hour* or time)).tw,kw,kf. | 9799 |
| 26 | hour* of work*.tw,kw,kf. | 3336 |
| 27 | (worktime adj2 arrangement*).tw,kw,kf. | 5 |
| 28 | (work* adj2 time adj2 arrangement*).tw,kw,kf. | 49 |
| 29 | (work* adj2 (duration* or night* or evening* or schedule* or weekend* or saturday* or sunday* or on call)).tw,kw,kf. | 5552 |
| 30 | ((three or two) adj (shift or shifts)).tw,kw,kf. | 388 |
| 31 | (compressed adj2 work*).tw,kw,kf. | 127 |
| 32 | quick shift chang*.tw,kw,kf. | 1 |
| 33 | (duty and (hour* adj3 (change* or restriction* or limit*))).tw,kw,kf. | 473 |
| 34 | or/1-33 | 34841 |
| 35 | (shift or shifts).tw,kw,kf. | 273189 |
| 36 | (quick adj (return* or change over* or changeover* or turn around or turnaround)).tw,kw,kf. | 314 |
| 37 | (short adj (off-duty or change over* or changeover* or turn around or turnaround or rest or break* or free time or freetime or recover*)).tw,kw,kf. | 906 |
| 38 | 36 or 37 | 1216 |
| 39 | 35 and 38 | 62 |
| 40 | 34 or 39 | 34859 |
| 41 | Patient Safety/ | 13530 |
| 42 | Patient Harm/ | 99 |
| 43 | Safety/ | 37394 |
| 44 | Medical Errors/ | 15023 |
| 45 | Medication Errors/ | 11989 |
| 46 | Diagnostic Errors/ | 34937 |
| 47 | Near Miss, Healthcare/ | 75 |
| 48 | Mortality/ | 38945 |
| 49 | Hospital Mortality/ | 32094 |
| 50 | fatal outcome/ | 58106 |
| 51 | Survival Rate/ | 151523 |
| 52 | Death/ | 16190 |
| 53 | Death, Sudden/ | 11829 |
| 54 | Patient Readmission/ | 12626 |
| 55 | Patient Discharge/ | 24584 |
| 56 | "Length of Stay"/ | 74570 |
| 57 | Inappropriate Prescribing/ | 2023 |
| 58 | Accidents/ | 18923 |
| 59 | Accident Prevention/ | 8846 |
| 60 | Accidental Falls/ | 20055 |
| 61 | Morbidity/ | 27294 |
| 62 | (patient* adj4 safet*).tw,kw,kf. | 39991 |
| 63 | (Patient* adj2 harm*).tw,kw,kf. | 3790 |
| 64 | (patient* adj3 outcome*).tw,kw,kf. | 190982 |
| 65 | safe practice*.tw,kw,kf. | 1134 |
| 66 | (safet* adj3 (event* or implication*)).tw,kw,kf. | 3436 |
| 67 | (adverse adj2 event*).tw,kw,kf. | 138149 |
| 68 | (mortalit* or morbidit* or (readmi* adj2 rate*) or length of stay or error* or near miss* or close call* or fatal outcome* or inappropriate prescri*).tw,kw,kf. | 1065390 |
| 69 | ((risk* or incident*) adj2 report*).tw,kw,kf. | 11790 |
| 70 | ((patient* or inpatient* or hospital* or untoward) adj3 incident*).tw,kw,kf. | 8772 |
| 71 | ((hospital* or patient* or inpatient* or risk* or rate*) adj3 complication*).tw,kw,kf. | 155006 |
| 72 | sentinel event*.tw,kw,kf. | 799 |
| 73 | or/41-72 | 1831813 |
| 74 | 40 and 73 | 4365 |
| 75 | (patient* or inpatient* or hospital* or nurse* or nursing or doctor* or physician* or surgeon* or resident* or midwi* or healthcare* or health care* or (medical adj (staff* or service*))).mp. | 7610662 |
| 76 | 74 and 75 | 3103 |
| 77 | limit 76 to (danish or english or norwegian or swedish) | 2896 |

**Database:** PsycINFO 1806 to January Week 3 2018

**Date:** 26 January 2018

**Number of results:** 427

| # | Searches | Results |
| --- | --- | --- |
| 1 | work scheduling/ | 1464 |
| 2 | Workday Shifts/ | 1856 |
| 3 | work week length/ | 142 |
| 4 | Work rest cycles/ | 222 |
| 5 | shiftwork*.mp. | 463 |
| 6 | nightwork*.mp. | 27 |
| 7 | nightshift*.mp. | 40 |
| 8 | (night* adj2 schedule*).mp. | 77 |
| 9 | (overtime adj3 (hour* or work*)).mp. | 270 |
| 10 | (flextime or flex time or flexitime or flexi time).mp. | 183 |
| 11 | (long* adj2 work* adj2 (hour* or week* or day* or period*)).mp. | 875 |
| 12 | (short* adj2 work* adj2 (hour* or week* or day* or period*)).mp. | 130 |
| 13 | ((long* or short*) adj2 (workweek* or workday*)).mp. | 36 |
| 14 | (flex* adj1 work*).mp. | 1125 |
| 15 | ((extend* or reduc*) adj3 (duty or duties or work*) adj3 (time or hour* or period* or week* or shift* or day* or schedule*)).mp. | 642 |
| 16 | ((extend* or reduc* or irregular*) adj2 (workweek* or workday*)).mp. | 27 |
| 17 | (irregular* adj3 (work* or shift or shifts)).mp. | 194 |
| 18 | ((unsocia* or antisocia* or anti socia*) adj2 (work* or shift or shifts)).mp. | 64 |
| 19 | ((nonstandard or non standard) adj2 (work* or shift or shifts)).mp. | 223 |
| 20 | ((shift or shifts) adj2 work*).mp. | 3213 |
| 21 | ((shift or shifts) adj2 (rota* or system or systems or schedule* or roster* or fixed or permanent)).mp. | 1001 |
| 22 | ((shift or shifts) adj2 (extend* or pattern* or cycle* or duration* or recover*)).mp. | 592 |
| 23 | ((shift or shifts) adj2 (evening or night* or late or early or weekend or twilight*)).mp. | 1156 |
| 24 | ((shift or shifts) adj2 (continental or continuous or turnaround or turn around or split)).mp. | 85 |
| 25 | ((shift or shifts) adj length*).mp. | 49 |
| 26 | (hour* adj (shift or shifts or duty or day or days or work* or week*)).mp. | 2222 |
| 27 | (workhour* or worktime).mp. | 47 |
| 28 | (work* adj (hour* or time)).mp. | 4010 |
| 29 | hour* of work*.mp. | 1967 |
| 30 | (worktime adj2 arrangement*).mp. | 1 |
| 31 | (work* adj2 time adj2 arrangement*).mp. | 69 |
| 32 | (work* adj2 (duration* or night* or evening* or schedule* or weekend* or saturday* or sunday* or on call)).mp. | 2798 |
| 33 | ((three or two) adj (shift or shifts)).mp. | 143 |
| 34 | (compressed adj2 work*).mp. | 56 |
| 35 | quick shift chang*.mp. | 1 |
| 36 | (duty and (hour* adj3 (change* or restriction* or limit*))).mp. | 72 |
| 37 | or/1-36 | 14252 |
| 38 | (shift or shifts).mp. | 67341 |
| 39 | (quick adj (return* or change over* or changeover* or turn around or turnaround)).mp. | 46 |
| 40 | (short adj (off-duty or change over* or changeover* or turn around or turnaround or rest or break* or free time or freetime or recover*)).mp. | 189 |
| 41 | 39 or 40 | 234 |
| 42 | 38 and 41 | 28 |
| 43 | 37 or 42 | 14258 |
| 44 | patient safety/ | 994 |
| 45 | safety/ | 11905 |
| 46 | errors/ | 9633 |
| 47 | mortality rate/ | 6575 |
| 48 | "death and dying"/ | 28271 |
| 49 | hospital discharge/ | 1930 |
| 50 | treatment duration/ | 3746 |
| 51 | accidents/ | 2362 |
| 52 | accident prevention/ | 1414 |
| 53 | falls/ | 2382 |
| 54 | morbidity/ | 5158 |
| 55 | (patient* adj4 safet*).mp. | 4848 |
| 56 | (patient* adj2 harm*).mp. | 883 |
| 57 | (patient* adj3 outcome*).mp. | 18463 |
| 58 | safe practice*.mp. | 200 |
| 59 | (safet* adj3 (event* or implication*)).mp. | 988 |
| 60 | (adverse adj2 event*).mp. | 11964 |
| 61 | (mortalit* or morbidit* or (readmi* adj2 rate*) or length of stay or error* or near miss* or close call* or fatal outcome* or inappropriate prescri*).mp. | 159243 |
| 62 | ((risk* or incident*) adj2 report*).mp. | 3955 |
| 63 | ((patient* or inpatient* or hospital* or untoward) adj3 incident*).mp. | 738 |
| 64 | ((hospital* or patient* or inpatient* or risk* or rate*) adj3 complication*).mp. | 2619 |
| 65 | sentinel event*.mp. | 116 |
| 66 | or/44-65 | 230127 |
| 67 | 43 and 66 | 1011 |
| 68 | (patient* or inpatient* or hospital* or nurse* or nursing or doctor* or physician* or surgeon* or resident* or midwi* or healthcare* or health care* or (medical adj (staff* or service*))).mp. | 955578 |
| 69 | 67 and 68 | 513 |
| 70 | limit 69 to (peer reviewed journal and (danish or english or norwegian or swedish)) | 427 |

**Database:** Cinahl with Full Text via EBSCOhost – Advanced search

**Date:** 26 January 2018

**Number of results:** 1007

| **#** | **Query** | **Limiters/Expanders** | **Results** |
| --- | --- | --- | --- |
| S1 | (MH "Flexible Scheduling") | Search modes - Boolean/Phrase | 1,005 |
| S2 | (MH "Shiftwork") | Search modes - Boolean/Phrase | 2,410 |
| S3 | (MH "Shift Workers") | Search modes - Boolean/Phrase | 1,135 |
| S4 | TI shiftwork* OR AB shiftwork* | Search modes - Boolean/Phrase | 210 |
| S5 | TI nightwork* OR AB nightwork* | Search modes - Boolean/Phrase | 5 |
| S6 | TI nightshift* OR AB nightshift* | Search modes - Boolean/Phrase | 38 |
| S7 | TI night* N2 schedule* OR AB night* N2 schedule* | Search modes - Boolean/Phrase | 42 |
| S8 | TI ( (overtime N3 (hour* OR work*)) ) OR AB ( (overtime N3 (hour* OR work*)) ) | Search modes - Boolean/Phrase | 297 |
| S9 | TI ( flextime OR "flex time" OR flexitime OR "flexi time" ) OR AB ( flextime OR "flex time" OR flexitime OR "flexi time" ) | Search modes - Boolean/Phrase | 41 |
| S10 | TI ( (long* N2 work* N2 (hour* or week* or day* or period*)) ) OR AB ( (long* N2 work* N2 (hour* or week* or day* or period*)) ) | Search modes - Boolean/Phrase | 482 |
| S11 | TI ( (short* N2 work* N2 (hour* or week* or day* or period*)) ) OR AB ( (short* N2 work* N2 (hour* or week* or day* or period*)) ) | Search modes - Boolean/Phrase | 83 |
| S12 | TI ( ((long* or short*) N2 (workweek* or workday*)) ) OR AB ( ((long* or short*) N2 (workweek* or workday*)) ) | Search modes - Boolean/Phrase | 15 |
| S13 | TI (flex* N1 work*) OR AB (flex* N1 work*) | Search modes - Boolean/Phrase | 639 |
| S14 | TI ( ((extend* or reduc*) N3 (duty or duties or work*) N3 (time or hour* or period* or week* or shift* or day* or schedule*)) ) OR AB ( ((extend* or reduc*) N3 (duty or duties or work*) N3 (time or hour* or period* or week* or shift* or day* or schedule*)) ) | Search modes - Boolean/Phrase | 540 |
| S15 | TI ( ((extend* or reduc* or irregular*) N2 (workweek* or workday*)) ) OR AB ( ((extend* or reduc* or irregular*) N2 (workweek* or workday*)) ) | Search modes - Boolean/Phrase | 20 |
| S16 | TI ( (irregular* N3 (work* or shift or shifts)) ) OR AB ( (irregular* N3 (work* or shift or shifts)) ) | Search modes - Boolean/Phrase | 79 |
| S17 | TI ( ((unsocia* or antisocia* or "anti socia*") N2 (work* or shift or shifts)) ) OR AB ( ((unsocia* or antisocia* or "anti socia*") N2 (work* or shift or shifts)) ) | Search modes - Boolean/Phrase | 36 |
| S18 | TI ( ((nonstandard or "non standard") N2 (work* or shift or shifts)) ) OR AB ( ((nonstandard or "non standard") N2 (work* or shift or shifts)) ) | Search modes - Boolean/Phrase | 39 |
| S19 | TI ( ((shift or shifts) N2 work*) ) OR AB ( ((shift or shifts) N2 work*) ) | Search modes - Boolean/Phrase | 1,885 |
| S20 | TI ( ((shift or shifts) N2 (rota* or system or systems or schedule* or roster* or fixed or permanent)) ) OR AB ( ((shift or shifts) N2 (rota* or system or systems or schedule* or roster* or fixed or permanent)) ) | Search modes - Boolean/Phrase | 615 |
| S21 | TI ( ((shift or shifts) N2 (extend* or pattern* or cycle* or duration* or recover*)) ) OR AB ( ((shift or shifts) N2 (extend* or pattern* or cycle* or duration* or recover*)) ) | Search modes - Boolean/Phrase | 382 |
| S22 | TI ( ((shift or shifts) N2 (evening or night* or late or early or weekend or twilight*)) ) OR AB ( ((shift or shifts) N2 (evening or night* or late or early or weekend or twilight*)) ) | Search modes - Boolean/Phrase | 1,173 |
| S23 | TI ( ((shift or shifts) N2 (continental or continuous or turnaround or "turn around" or split)) ) OR AB ( ((shift or shifts) N2 (continental or continuous or turnaround or "turn around" or split)) ) | Search modes - Boolean/Phrase | 35 |
| S24 | TI ( ((shift or shifts) N1 length*) ) OR AB ( ((shift or shifts) N1 length*) ) | Search modes - Boolean/Phrase | 97 |
| S25 | TI ( (hour* W1 (shift or shifts or duty or day or days or work* or week*)) ) OR AB ( (hour* W1 (shift or shifts or duty or day or days or work* or week*)) ) | Search modes - Boolean/Phrase | 5,093 |
| S26 | TI ( (workhour* or worktime) ) OR AB ( (workhour* or worktime) ) | Search modes - Boolean/Phrase | 18 |
| S27 | TI ( ("work* hour*" or "work* time") ) OR AB ( ("work* hour*" or "work* time") ) | Search modes - Boolean/Phrase | 2,489 |
| S28 | TI hour* W1 work* OR AB hour* W1 work* | Search modes - Boolean/Phrase | 1,082 |
| S29 | TI (worktime N2 arrangement*) OR AB (worktime N2 arrangement*) | Search modes - Boolean/Phrase | 0 |
| S30 | TI (work* N2 time N2 arrangement*) OR AB (work* N2 time N2 arrangement*) | Search modes - Boolean/Phrase | 10 |
| S31 | TI ( (work* N2 (duration* or night* or evening* or schedule* or weekend* or saturday* or sunday* or "on call")) ) OR AB ( (work* N2 (duration* or night* or evening* or schedule* or weekend* or saturday* or sunday* or "on call")) ) | Search modes - Boolean/Phrase | 2,044 |
| S32 | TI ( ((three or two) W1 (shift or shifts)) ) OR AB ( ((three or two) W1 (shift or shifts)) ) | Search modes - Boolean/Phrase | 261 |
| S33 | TI (compressed N2 work*) OR AB (compressed N2 work*) | Search modes - Boolean/Phrase | 19 |
| S34 | TI "quick shift chang*" OR AB "quick shift chang*" | Search modes - Boolean/Phrase | 0 |
| S35 | TI ( (duty and (hour* N3 (change* or restriction* or limit*))) ) OR AB ( (duty and (hour* N3 (change* or restriction* or limit*))) ) | Search modes - Boolean/Phrase | 102 |
| S36 | S1 OR S2 OR S3 OR S4 OR S5 OR S6 OR S7 OR S8 OR S9 OR S10 OR S11 OR S12 OR S13 OR S14 OR S15 OR S16 OR S17 OR S18 OR S19 OR S20 OR S21 OR S22 OR S23 OR S24 OR S25 OR S26 OR S27 OR S28 OR S29 OR S30 OR S31 OR S32 OR S33 OR S34 OR S35 | Search modes - Boolean/Phrase | 15,221 |
| S37 | TI ( (shift or shifts) ) OR AB ( (shift or shifts) ) | Search modes - Boolean/Phrase | 20,896 |
| S38 | TI ( (quick N1 (return* or "change over*" or changeover* or "turn around" or turnaround)) ) OR AB ( (quick N1 (return* or "change over*" or changeover* or "turn around" or turnaround)) ) | Search modes - Boolean/Phrase | 66 |
| S39 | TI ( (short N1 ("off-duty" or "change over*" or changeover* or "turn around" or turnaround or rest or break* or "free time" or freetime or recover*)) ) OR AB ( (short N1 ("off-duty" or "change over*" or changeover* or "turn around" or turnaround or rest or break* or "free time" or freetime or recover*)) ) | Search modes - Boolean/Phrase | 360 |
| S40 | S38 OR S39 | Search modes - Boolean/Phrase | 424 |
| S41 | S37 AND S40 | Search modes - Boolean/Phrase | 19 |
| S42 | S36 OR S41 | Search modes - Boolean/Phrase | 15,228 |
| S43 | (MH "Patient Safety") | Search modes - Boolean/Phrase | 34,539 |
| S44 | (MH "Adverse Health Care Event") | Search modes - Boolean/Phrase | 4,151 |
| S45 | (MH "Adverse Drug Event") | Search modes - Boolean/Phrase | 5,258 |
| S46 | (MH "Medication Errors") | Search modes - Boolean/Phrase | 9,404 |
| S47 | (MH "Inappropriate Prescribing") | Search modes - Boolean/Phrase | 885 |
| S48 | (MH "Diagnostic Errors") | Search modes - Boolean/Phrase | 5,880 |
| S49 | (MH "Treatment Errors") | Search modes - Boolean/Phrase | 5,128 |
| S50 | (MH "Safety") | Search modes - Boolean/Phrase | 13,464 |
| S51 | (MH "Mortality") | Search modes - Boolean/Phrase | 17,019 |
| S52 | (MH "Hospital Mortality") | Search modes - Boolean/Phrase | 8,109 |
| S53 | (MH "Fatal Outcome") | Search modes - Boolean/Phrase | 3,515 |
| S54 | (MH "Death") | Search modes - Boolean/Phrase | 10,725 |
| S55 | (MH "Death, Sudden") | Search modes - Boolean/Phrase | 1,353 |
| S56 | (MH "Readmission") | Search modes - Boolean/Phrase | 6,662 |
| S57 | (MH "Patient Discharge") | Search modes - Boolean/Phrase | 9,727 |
| S58 | (MH "Length of Stay") | Search modes - Boolean/Phrase | 21,153 |
| S59 | (MH "Accidents") | Search modes - Boolean/Phrase | 1,740 |
| S60 | (MH "Accidental Falls") | Search modes - Boolean/Phrase | 13,309 |
| S61 | (MH "Morbidity") | Search modes - Boolean/Phrase | 4,919 |
| S62 | (MH "Sentinel Event") | Search modes - Boolean/Phrase | 912 |
| S63 | TI (patient* N3 safet*) OR AB (patient* N3 safet*) | Search modes - Boolean/Phrase | 16,690 |
| S64 | TI (Patient* N1 harm*) OR AB (Patient* N1 harm*) | Search modes - Boolean/Phrase | 1,428 |
| S65 | TI (patient* N2 outcome*) OR AB (patient* N2 outcome*) | Search modes - Boolean/Phrase | 41,271 |
| S66 | TI "safe practice*" OR AB "safe practice*" | Search modes - Boolean/Phrase | 661 |
| S67 | TI ( (safet* N2 (event* or implication*)) ) OR AB ( (safet* N2 (event* or implication*)) ) | Search modes - Boolean/Phrase | 966 |
| S68 | TI (adverse N1 event*) OR AB (adverse N1 event*) | Search modes - Boolean/Phrase | 26,797 |
| S69 | TI ( (mortalit* or morbidit* or (readmi* N1 rate*) or "length of stay" or error* or "near miss*" or "close call*" or "fatal outcome*" or "inappropriate prescri*") ) OR AB ( (mortalit* or morbidit* or (readmi* N1 rate*) or "length of stay" or error* or "near miss*" or "close call*" or "fatal outcome*" or "inappropriate prescri*") ) | Search modes - Boolean/Phrase | 137,978 |
| S70 | TI ( ((risk* or incident*) N1 report*) ) OR AB ( ((risk* or incident*) N1 report*) ) | Search modes - Boolean/Phrase | 3,264 |
| S71 | TI ( ((patient* or inpatient* or hospital* or untoward) N2 incident*) ) OR AB ( ((patient* or inpatient* or hospital* or untoward) N2 incident*) ) | Search modes - Boolean/Phrase | 1,613 |
| S72 | TI ( ((hospital* or patient* or inpatient* or risk* or rate*) N2 complication*) ) OR AB ( ((hospital* or patient* or inpatient* or risk* or rate*) N2 complication*) ) | Search modes - Boolean/Phrase | 16,678 |
| S73 | S43 OR S44 OR S45 OR S46 OR S47 OR S48 OR S49 OR S50 OR S51 OR S52 OR S53 OR S54 OR S55 OR S56 OR S57 OR S58 OR S59 OR S60 OR S61 OR S62 OR S63 OR S64 OR S65 OR S66 OR S67 OR S68 OR S69 OR S70 OR S71 OR S72 | Search modes - Boolean/Phrase | 326,506 |
| S74 | S42 AND S73 | Search modes - Boolean/Phrase | 2,023 |
| S75 | TI ( (patient* or inpatient* or hospital* or nurse* or nursing or doctor* or physician* or surgeon* or resident* or midwi* or healthcare* or "health care*" or "medical staff*" or "medical service*") ) OR AB ( (patient* or inpatient* or hospital* or nurse* or nursing or doctor* or physician* or surgeon* or resident* or midwi* or healthcare* or "health care*" or "medical staff*" or "medical service*") ) OR MW ( (patient* or inpatient* or hospital* or nurse* or nursing or doctor* or physician* or surgeon* or resident* or midwi* or healthcare* or "health care*" or "medical staff*" or "medical service*") ) | Search modes - Boolean/Phrase | 1,820,708 |
| S76 | S74 AND S75 | Search modes - Boolean/Phrase | 1,763 |
| S77 | S74 AND S75 | Limiters - Research Article; Language: Danish, English, Norwegian, Swedish  Search modes - Boolean/Phrase | 1,007 |

**Database:** Web of Science Core Collection: Citation Indexes

Science Citation Index Expanded (SCI-EXPANDED) --1987-present

Social Sciences Citation Index (SSCI) --1987-present

Arts & Humanities Citation Index (A&HCI) --1987-present

Emerging Sources Citation Index (ESCI) --2015-present

**Date:** 25 January 2018

**Number of results:** 2600

| # 26 | 2,600 | (#25 AND #24) AND LANGUAGE: (English OR Danish OR Norwegian OR Swedish) AND DOCUMENT TYPES: (Article OR Review) |
| --- | --- | --- |
|  |  | Indexes=SCI-EXPANDED, SSCI, A&HCI, ESCI Timespan=1987-2018 |
| # 25 | 5,672,674 | TS=(patient* or inpatient* or hospital* or nurse* or nursing or doctor* or physician* or healthcare* or “health care*” or “medical staff*” or resident* or surgeon* or midwi* or “medical service*”) |
| # 24 | 5,014 | #23 AND #13 |
| # 23 | 1,950,259 | #22 OR #21 OR #20 OR #19 OR #18 OR #17 OR #16 OR #15 OR #14 |
| # 22 | 183,785 | TS=( patient* NEAR/2 outcome*) |
| # 21 | 118,483 | TS=((hospital* or patient* or inpatient* or risk* or rate*) NEAR/2 complication*) |
| # 20 | 7,893 | TS= ((patient* or inpatient* or hospital* or untoward) NEAR/2 incident*) |
| # 19 | 11,075 | TS= ((risk* or incident*) NEAR/1 report*) |
| # 18 | 1,598,955 | TS= ( (readmi* NEAR/1 rate*) OR “length of stay” OR “near miss*” OR “close call*” OR “fatal outcome*” OR “Inappropriate prescri*” OR “Survival rate” OR error* OR Mortali* OR Morbidit* OR "sentinel event*" ) |
| # 17 | 133,644 | TS=(adverse NEAR/1 event*) |
| # 16 | 4,04 | TS=(safet* NEAR/2 (event* or implication*)) |
| # 15 | 2,851 | TS= (Patient* NEAR/1 harm*) |
| # 14 | 35,845 | TS=(patient* NEAR/3 safet*) |
| # 13 | 48,204 | #12 OR #9 |
| # 12 | 99 | #11 AND #10 |
| # 11 | 2,719 | TS=((quick NEAR/0 (return* OR "change over*" OR changeover* OR "turn around" OR turnaround)) OR (short NEAR/0 ("off-duty" OR "change over*" OR changeover* OR "turn around" OR turnaround OR rest OR break* OR "free time" OR freetime OR recover*))) |
| # 10 | 719,067 | TS=(shift OR shifts) |
| # 9 | 48,144 | #8 OR #7 OR #6 OR #5 OR #4 OR #3 OR #2 OR #1 |
| # 8 | 507 | TS=((duty AND (hour* NEAR/2 (change* or restriction* or limit*)))) |
| # 7 | 7,25 | TS=((worktime NEAR/1 arrangement*) OR (work* NEAR/1 time NEAR/1 arrangement*) OR (work* NEAR/1 (duration* OR night* OR evening* OR schedule* OR weekend* OR saturday* OR sunday* OR "on call")) OR ((three or two) NEAR/0 (shift or shifts)) OR (compressed NEAR/1 work*)) |
| # 6 | 18,281 | TS=((hour* NEAR/0 (shift OR shifts OR duty OR day OR days OR work* OR week*)) OR (work* NEAR/0 (hour* OR time))) |
| # 5 | 20,705 | TS=(((shift OR shifts) NEAR/1 (work* OR rota* OR system OR systems OR schedule* OR roster* OR fixed OR permanent OR extend* OR pattern* OR cycle* OR duration* OR recover* OR evening OR night* OR late OR early OR weekend OR twilight* OR continental OR continuous OR turnaround OR "turn around" OR split)) OR ((shift OR shifts) NEAR/0 length*)) |
| # 4 | 3,286 | TS=(((extend* OR reduc*) NEAR/2 (duty OR duties OR work*) NEAR/2 (time OR hour* OR period* OR week* OR shift* OR day* OR schedule*)) OR ((extend* OR reduc* OR irregular*) NEAR/1 (workweek* OR workday*)) OR (irregular* NEAR/2 (work* OR shift OR shifts)) OR ((unsocia* OR antisocia* OR "anti socia*") NEAR/1 (work* OR shift OR shifts)) OR ((nonstandard OR "non standard") NEAR/1 (work* OR shift OR shifts))) |
| # 3 | 1,903 | TS=((long* NEAR/1 work* NEAR/1 (hour* OR week* OR day* OR period*)) OR (short* NEAR/1 work* NEAR/1 (hour* OR week* OR day* OR period*)) OR ((long* OR short*) NEAR/1 (workweek* OR workday*))) |
| # 2 | 4,119 | TS= ((night* NEAR/1 schedule*) OR (overtime NEAR/2 (hour* OR work*)) OR (flextime OR "flex time" OR flexitime OR "flexi time") OR (flex* NEAR/1 work*)) |
| # 1 | 2,129 | TS=(shiftwork* OR nightwork* OR nightshift* OR workhour* OR worktime OR "hour* of work*" OR "hour* at work*" OR "quick shift change*") |
