# Supplementary material for: Healthy working time arrangements for healthcare personnel and patients: a systematic literature review
Source: BMC Health Serv Res. 2019 Mar 27;19:193. doi: 10.1186/s12913-019-3993-5 (PMC6437911; doi:10.1186/s12913-019-3993-5)
Supplement: Supplementary file 2 — Appendix 2. Table of studies. Table of data extracted from included studies. (DOCX 160 kb) [file 12913_2019_3993_MOESM2_ESM.docx]

## Attachment 2: Included studies

##

| **Study** | **Working time arrangement** | **Employee health** | **Patient safety** | **Selection (size and response rate)** | **Control variables** | **Study design** | **Findings** |
| --- | --- | --- | --- | --- | --- | --- | --- |
| Admi, Tzischinsky, Epstein, Herer, and Lavie (2008) | Shift work (rotating three-shift arrangement, 8 hours per shift) compared with day shift | Health issues (high blood pressure, diabetes and leg pains, among others) Sleep (SDQ) Self-reported | Errors and incidents. Retrieved from hospital database. | Nurses in a hospital in Israel (N=699, response rate 93) | Age, BMI and gender. | Cross-sectional | Day shift workers more frequently reported a series of health issues (e.g. high blood pressure, digestive illnesses, diabetes), but these were no longer significant when controlled for age, BMI and gender. Shift workers reported significantly more frequently on problems sleeping, also after inclusion of control variables (p<0.05). Non-adaptive shift workers (shift workers who often reported having problems sleeping and who woke several times a night) – reported errors insignificantly more frequently (p=0.14). |
| Ali et al. (2011) | Intensive care doctors worked in the control group at the intensive care unit for 14 days consecutively, with on-call duty at night. The experiment group had Saturdays and Sundays off during the same period, while another doctor from the group took the weekend shifts. | Burnout  Self-reported. | Length of stay at the intensive care unit and hospital, mortality at the intensive care unit and hospital. Hospital data. | IC doctors at five intensive care units in the USA, (N= 45 doctors total, but 39 for burnout). 1,900 patients. | Patient demography (age, gender, ethnic background), stay prior to intensive care unit, severity of illness, workload at the unit, unit, time. | Prospective group-randomized study | A non-significant improvement in patient outcomes for the experiment group after control variables were added to the model (length of stay b 0.36 p 0.20, and 0.34 p 0.71; mortality OR 1.43 p 0.12, and OR 1.17 p 0.41). Significantly higher burnout in the control group (b 2.77 p 0.003). |
| Amirian, Andersen, Rosenberg, and Gögenur (2014) | Test of skills and sleepiness before and after 17-hour night shift.  Sleep was allowed during the shift if possible. | Sleepiness (KSS). Self-reported.  Sleep measured with wristband. | Job performance and error in a surgical simulator | Danish surgeons at various levels (N=29) | Professional level | Surgeons’ performance was measured before and after the shift. (Wilcoxon test and Friedman test for repeated measurements) | No significant differences in performance in the simulator before and during the shift. There was a significant increase in sleepiness during the night shift, but also significant improvement on cognitive tests. The surgeons slept significantly less the nights they worked night shift. |
| Amirian, A. K. Danielsen, and Rosenberg (2013) |  |  |  | Surgeons (N=13) |  | Qualitative | The doctors described fatigue as a natural consequence of working night shifts, including a feeling of hangover, nausea and problems sleeping for some, and challenges with near traffic accidents on the way home for many.  Several surgeons told of how the fatigue led to reduced mental capacity and a particular lack of concentration skills.  The surgeons consistently reported reduced communication skills when they were fatigued.  The problems were particularly tied to routine work that did not provide them with adrenaline to keep them awake. |
| Arakawa, Kanoya, and Sato (2011) | Breaks during the night shift, number of night shifts per month, frequency of overtime on the night shift. | Satisfaction with amount of sleep, limitations in daily life due to sleep deprivation, mental health, pain, general health, vitality, treatment for illness, sick leave the past six months.  Self-reported. | Errors.  Self-reported | Nurses at 93 hospitals in Japan (N = 6 445, response rate 78.6). | Age, work experience, workload, number of staff, stress, quality of life (physical and social functional skills, physical and emotional role) civil status, children, type of workplace | Cross-sectional, logistical regression. | Nurses experienced errors more frequently if they had been on sick leave during the past six months (OR 1.50 p<0.001), were undergoing treatment for illness (OR 1.21 p>0.05), experienced bodily pain (OR 0.995 p<0.05) and had more breaks during the night shifts (OR 1.003 p<0.01). Frequent overtime and several night shifts per month were related to more perceived errors in a simple correlation analysis, but the correlation was no longer significant when health and other control variables were controlled for. |
| Arimura, Imai, Okawa, Fujimura, and Yamada (2010) | Day shift workers or shift workers (rotating two and three-shift arrangements). Overtime and frequency of evening and night shifts for shift workers. | Mental health (GHQ), sleepiness (ESS), sleep quality (PSQI).  Self-reported. | Medical error. Self-reported | Nurses at two hospitals in Japan (N= 454, response rate 77.9). | Time off, sleep requirements, department and «too much work». | Cross-sectional, logistical regression. | Reduced mental health (OR 1.1 CI 1.0-1.1) and shift work (2.1 1.2-3.9) were both significantly related to increased errors after having controlled for control variables. Sleepiness and reduced sleep quality were related to increased probability of error in a simple correlation analysis, but not after having controlled for control variables, including mental health and shift work. Overtime and frequency of evening and night shifts for shift workers was not significantly related to errors in a simple correlation analysis (t-test). |
| Arzalier-Daret et al. (2017) | Night shifts. Performance was measured at 9:00 a.m. after a night off and after a night shift. | Sleep. Objectively measured with wristband. | Execution of a simulated medical crisis. Examples include administration of medicine the patient is allergic to and stopping of anaesthetic medicine after the patient has gone into anaphylactic shock. Evaluated by a blind third party. | Anaesthetists specializing in France (N=48). | None. | Each doctor was measured both after a night off and after a night shift, each doctor was his own control. The doctors were randomized in two different simulator tests. | The doctors had slept significantly less after a night shift (2 hours) than after a night off (7 hours, p < 0.001). The doctors who had worked night shift also performed the simulator test significantly more poorly (p>0.05). There were no significant differences in the ability to control equipment before starting. |
| Asaoka et al. (2013) | Two or three-shift rotations (both with nights of 8 or 17 hours, respectively), working several night shifts consecutively, possibility for rest during shifts, number of hours at work, number of hours of night shift | Insomnia or severe sleepiness tied to shift work (shift work disorder -SWD) and depressive symptoms (CES-D). Self-reported. | Error and near-error (procedural error and medication error). Self-reported. | Female nurses on rotating shifts with nights in two hospitals in Japan. (N= 997, response rate 80.5). | Family structure, leadership responsibility, work experience, A/B-person. | Cross-sectional. Chi-square test (no control variables) for correlation between SWD and errors. Logistical regression for working time and SWD. | Nurses with SWD had significantly greater probability of procedural and medical near-error (p < 0.001 and actual procedural error (p < 0.001). All working time variables were significantly related to SWD before adding control variables. No possibility for a rest and several hours of night work were significantly related to increased risk of SWD also after adding control variables (respectively, OR 1.97 p<0.01 and OR 2.23 p<0.05) |
| Bae (2013) | Imposed overtime and regulation of such. Regulation means that nurses may say no to working more than 12 hours per day (compared two states, with and without regulation), amount of actual weekly working time | Needle stick (self-imposed), pressure and sprains, cuts and cut wounds, bruises. Self-reported. | Medication errors, falls, pressure sores, infections, not able to save the patient.  Self-reported. | Nurses in two American states (N= 173, response rate 29.8). | Type of hospital and department, workload, educational level | Cross-sectional, logistical regression. | Regulation of imposed overtime did not have effect on nurses’ health, nurses with on-call duty had increased collective risk of nurse injuries (OR 3.93 (1.47 – 10.50)). Regulation gave higher risk of adverse patient incidents (falls, pressure sores and infections). Working more than 40 hrs per week significantly increased the chance of medication errors, falls and pressure sores, but not employee injuries (needle stick (self-imposed), pressure and sprains, cuts and cut injuries, bruises). |
| Balch et al. (2011) | Hours per week, nights on call per week | Burnout (MBI), depression (PRIME MD), suicidal thoughts. Self-reported. | Sued for malpractice. Self-reported. | Surgeons in the USA – members of the American College of Surgeons (N=7 164, response rate 29). | Controlled for «*other personal and professional characteristics*» in parts of the multivariate analysis. | Cross-sectional, linear and logistical regression | Doctors who had been sued in the past 24 months had higher risk of burnout (OR 1.39, p>0.0001), symptoms of depression (OR 1.49, p <0.0001) and suicidal thoughts (OR 1.64, p <0.0001). The lawsuit also had correlation with number of hours per week (1 extra hour gave OR 1.006 (1.002-1.009)), and number of nights on call per week (one extra on-call gave OR 1.039 (1.009-1.069)). |
| Baldwin (2003)  (Baldwin, Daugherty, Tsai, & Scotti, 2003) | Hours worked per week (on average) | Sleep, accident, injury, alcohol use.  Self-reported. | Considerable medical error.  Self-reported. | Specialist doctors in the USA (residents) (N=3604 response rate 64.2). | Year of residency, gender, children, civil status, country of education, ethnic background, adopted, death of close friend/family, divorced, work conflicts, medication, demand for immoral or unethical treatment. | Cross-sectional. Logistical regression. | Logistical regression showed that doctors who worked more than 80 hours had increased odds of accident or injury (OR 1.58 CI 1.08-2.30), increased alcohol use (OR 1.62 CI 1.23-2.16), and weight change (OR 1.63 CI 1.42-1.88). They also had increased odds of making a considerable medical error due to sleep deprivation (OR 1.54 CI 1.33-1.79) and making a medical error that resulted in a negative patient outcome (OR 1.62 CI1.18-2.20). Simple correlation analysis (no control variables) also showed that number of hours worked per week was significantly related to hours of sleep (-.39), most number of hours without sleep (.31) and frequency of extended sleep deprivation (.42). |
| Baldwin and Daugherty (2004)  Same data as Baldwin (2003) | Hours worked per week (on average) | Sleep.  Self-reported. | Considerable medical error.  Self-reported. | Specialist doctors in the USA (residents) (N=3 604 response rate 64.2). | None (work hours in an additional analysis). | Cross-sectional. Regression analysis. | Number of hours worked per week was significantly related to hours of sleep (-.39). Stepwise multiple regression analysis showed that increased explained variance in sleep was low (6 percent). Hours of sleep was also significantly correlated with having made medical errors (p<0.001), being involved in a malpractice lawsuit (p<0.05), alcohol use (p<0.001), weight change (p<0.001) and injury/accident (p<0.001).  Sleep was also significantly related to error when the authors controlled for work hours, but the correlation between work hours and errors is not shown in the article. |
| Barger et al. (2006) | Long shifts (>24timer) (0, 1-4 times and 5 or more times per month).  Self-reported. | Dozed off at work. Self-reported. | Fatigue-related medical errors and other medical errors. Whether or not the errors led to preventable negative patient outcomes and death.  Self-reported. | Interns, USA  (N=2 737, response rate 15). | Individuals were their own control. | Longitudinal study. Dependent and independent variables measured once a month. | For the months staff worked long shifts (1-4 and 5 or more long shifts) odds were significantly increased for fatigue-related error (OR 3.5 CI 3.3-3.7) from 3.8 percent probability of error in months with no long shifts to 16 percent probability of error in months with five or more long shifts. The odds of a fatigue-related error that resulted in preventable negative patient outcome were also significantly higher (OR 8.7 CI3.4-22 and OR7.0 CI 4.3-11). Fatigue-related errors that resulted in the patient’s death were significantly higher in months with five or more long shifts (OR 4.1 CI 1.4- 12). There was also a signigicant but moderate increase in non-fatigue-related errors. The doctors also slept fewer hours per month the more long shifts they worked (significance not provided) and the odds for dozing off during surgery (OR 2.1 CI 1.7-2.7 and OR 1.4 CI1.3-1.6), during patient examinations (OR 1.5 CI 1.3-1.7 and OR 2.1 CI 2.0-2.2) and during educational activities such as lectures (OR 1.99 CI1.96-2.03 and OR 4.3 CI4.3-4.4) increased significantly. |
| Bilimoria et al. (2016) | Shift arrangements that are normal according to the U.S. Accreditation Council for Graduate Medical Education (ACGME) duty-hour policies (80/16) (standard) versus arrangements that are more flexible with regard to the 16-hour requirement and the requirement of rest between shifts, while upholding the prohibition of more than 80 hours per week (flexible). | Well-being, fatigue,  Self-reported. | Mortality, serious complications, complications, sepsis, surgical infection, and urinary tract infection, among others. Patient journals (ACS NSQIP)  Fatigue effect on patient safety. Self-reported | American doctors in training (N=4330 doctors, N=  138 691 patients) | Patient characteristics | Group-randomized intervention | No significant differences in objectively measured patient outcomes (patient journals). The doctors in the standard group were less satisfied with the quality of patient treatment and more inclined to believe that work hour regulations negatively affected patient safety (self-reported). No significant differences in general well-being of the doctors. No significant differences in whether the doctors felt that fatigue negatively impacted patient safety or their own safety. |
| Cammu and Haentjens (2012) | Work hours per week (>41 hours, 41-60 hours, 61-80 hours, >80 hours) | Fatigue, car accident or near accident due to fatigue.  Self-reported. | Medical or surgical error due to fatigue  Self-reported. | Gynecologists in Belgium (N=260, response rate 58). | Age, workplace, sporting activity, satisfied with own work, calls at night | Cross-sectional. Logistical regression. | Working 61-80 hours a week (OR 9.9 CI1.1-93.4) and more than 80 hours a week (OR 15.2 CI 1.3-175.2) were related to fatigue. In all, 15 percent of the doctors reported having made a medical error due to fatigue, and 5 percent having made a medical error. The most common mistake was connected to medication (e.g. incorrect dose, incorrect medicine), but the doctors also reported errors such as unnecessary cesarean, unnecessary removal of ovaries and perforation of the uterus. The authors did not test the correlations between errors and work hours or fatigue measurements. |
| Cappuccio (2009)  Cappuccio et al. (2009) | Work hours per week <48 hours (intervention) and <56 hour (on average)  The intervention was an introduction of the «European work hours directive”, but also included other measures  (the intervention was planned for forward rotations, with maximum three consecutive night shifts and maximum 12 hours per shift. The doctors in the intervention also received advice on sleep hygiene.) | Sleep. Self-reported (daily for 12 weeks). | Negative incidents (a case where the patient treatment had negative consequences for the patient). And errors (that could have negative patient consequences). Taken from patient journals. | Junior doctors at two hospitals in England (N=19 doctors, N=481 patients). | None. | Intervention. Four groups of doctors were randomly divided into rotations of >56 hours and >48 hours. Patient outcomes were inspected for two of the groups. | The doctors in the intervention group worked nine hours less per week (p<0.001), and they slept on average 30 min more per day (p=0.095) –the difference in sleep was not significant. There was 33 percent fewer medical errors in the intervention group (HR 0.62 p<0.05), 31 percent fewer negative incidents (HR 0.63 p<0.05) and 83 percent fewer prevented but potentially negative incidents (HR 0.16 p<0.05). The doctors themselves reported concerns about the significance for their education. |
| Chen, Vorona, Chiu, and Ware (2008) | Work hours per week (continually from <30 to >100 hours). Self-reported. | Sleepiness (ESS) (probability of dozing off in various scenarios). Self-reported | Errors.  Self-reported | Doctors in the USA (N= 180, response rate 45). | Age, gender, private sector, work experience, surgery. | Cross-sectional. Linear regression. | They found no correlation between number of work hours per week and sleepiness (b 0-03 P=0.24). Significant correlation between reported sleepiness and a collective measurement on self-reported negative consequences of sleepiness. Of individual questions tied to error there was a significant positive correlation between sleepiness and having written an incorrect order due to sleepiness (p<0.05), and a non-significant positive correlation between sleepiness and having made a medical error due to sleep deprivation and fatigue (p =0.06). There was a weak and non-significant negative correlation between sleepiness and having written incorrect prescriptions after having been on call (p=0.32). |
| Chen et al. (2013) | Shift length, hours per week, number of times on call per week. | Burnout (MBI); emotional fatigue (EE) depersonalization (DP) and self-perceived job performance (SJ). Self-reported. | Experience with medical malpractice.  Self-reported | Doctors in Taiwan (N=839, response rate 76) | Alcohol consumption, satisfied with specialty, satisfied with patient-doctor relations. | Cross-sectional. Logistical regression. | Doctors who had worked 18 consecutive hours or more had 14.7 times as high risk of emotional burnout on a low level, and 2.8 times as high risk of burnout on a moderate level in relation to those who had worked 3-7 hours consecutively (p=0.05 and 0.268). Those who worked 65 or more hours per week had 1.4 times higher risk of burnout at a low level and 1.5 times higher risk of moderate burnout, both viewed in relation to those who worked 49-56 hours a week (p=0.002 and 0.009).  Doctors with experience of medical malpractice also had higher burnout levels.  Attn: There is not full conformity between the results in the text presented on page 1473 and table 4, page 1476. We have held to the text. |
| Dean, Scott, and Rogers (2006) |  |  |  | Nurses at a neonatal critical care unit in the USA (N=6). |  | Case study. In the course of 28 days the six nurses registered six errors. For the two errors that could have been partially due to fatigue, the nurses’ journals were inspected more closely. | *Error 1: medication administered at incorrect intervals.*  The nurse aged 55 was in the midst of a 17-hour night shift. It was her third consecutive night shift. She had only slept four hours in the past 24 hours and 12 hours in the past three days. She had worked 43 hours in the past three days. She reported difficulties keeping herself awake during the shift in which the error occurred.  *Error 2: The nurse aged 54 stopped herself just as she was about to give a deadly dose of morphine.*  The near-error occurred in the course of a 15-hour night shift. She had only had one 30-minute break during the shift. Before the night shift she had had several days off. She had slept five hours in the past 24 hours and reported difficulties keeping herself awake during the shift. |
| de Oliveira et al. (2013) | Work hours per week (>70 hours) and frequency of being called in (five days, or more or less than five days between call-ins). | Burnout (MBI), risk of depression (HANDS). Self-reported. | Self-reported errors with and without negative patient consequences, and self-reported medication errors. Self-reported. | Anaesthetists in training (residents) in the USA (N=1508, response rate 54). | None. | Cross-sectional. Binary logistical regression. | Working more than 70 hours per week and less frequent call-ins were significantly related to higher risk of burnout and depression. Self-reported errors with and without negative patient outcomes, and self-reported medication errors, were significantly high for doctors with both high burnout and depression, compared with those who have lower risk of both. All three types of error were also significantly higher for doctors with high risk of burnout than for those with low risk of burnout and depression. |
| Domen, Connelly and Spence (2015) | During on-call duty, number of call-ins per month and number of days worked per week | Fatigue during call. | Errors due to fatigue.  Self-reported. | Anaesthesiology nurses in the USA (N=325, response rate 13). | None. | Cross-sectional. | More work days per week (p 0.001), number of call-ins per month (p 0.001) and length of each weekend call-in (p 0.02) were significantly positively related to fatigue upon call-in. Length of everyday call-in was not significantly negatively related to fatigue on call (p = 0.08). 28 percent reported having made mistakes in patient treatment due to fatigue. The authors did not test the correlations between errors and work hours or fatigue measurements. |
| Dorrian et al. (2006) | Shift type (day, evening, night) and shift length | Sleep (length and quality).  Self-reported. | Errors (own and others’). Errors included medication, prescriptions, journal entries, following of procedures, patient count and other.  Self-reported. | Nurses in Australia (N=23). | None. | Diary study (daily measurements for a month) | Staff slept significantly longer on days with evening shifts than on days with day or night shifts. No significant correlation between shift length and length of sleep.  No significant correlation between shift type and shift length, on one hand, and own mistakes or noticing others’ mistakes on the other. Length of sleep was significantly negatively related to number of reported errors and near errors (B= -0.319 p>0.01). One extra hour of sleep reduced the rate of error by approximately 27 percent. Sleep length was not significantly positively related to noticing others’ mistakes (p=0.08) |
| Dorrian et al. (2008)  Partially the same selection as for Dorrian et al. (2006) | Shift length and number of consecutive shifts. | Length of sleep, struggling to stay awake, and extreme sleepiness.  Self-reported | Errors (own and others’).  Self-reported | Nurses in Australia (N=41). | None. | Diary study (daily measurements for a month) | Shift length (OR 1.5 CI 1.2-1.7) and sleep the previous 24 hours (OR 0.9 CI 0.9-1.0) were significantly related to struggling to stay awake. Number of consecutive shifts was significantly related to extreme sleepiness (OR 1.2 CI1.0-1.5). Struggling to stay awake was also significantly related to reported errors (OR 2.4 CI 1.1-4.9). |
| Eastridge et al. (2003) | 24-hour shifts (time point before, during and after the shift, previous work in the week) | Sleep patterns, fatigue.  Self-reported. | Job performance/ errors in a surgical simulator | American surgeons. Voluntary recruitment (N=35). | Number of years of training after primary education | Each surgeon was tested three times; two times before or at the start of a 24-hour shift (rested category) and the morning after a 24-hour shift (sleep deprivation category).  Each surgeon was his/her own control. | Before the shift, number of hours of sleep the previous 24 hours was approx. 6.5, versus approximately 1.5 after the shift (p<0.01). Subjective perception of fatigue rose from about 2.5 to almost 7 from before the shift to after the shift (on a scale from 1 to 10; p<0.001). Significant increase in the number of errors after the shift (from scarcely six to scarcely 12 errors) in relation to before the shift (p<0.01). Small changes in effectiveness |
| Estryn-Behar, Van der Heijden, and Grp (2012) | Shift type (day, night or rotating) and shift length (8, 10 or 12 hours) | Burnout, work ability, sick leave.  Self-reported | Frequency of concern for making an error. Self-reported | Nurses in the EU (N = 25 924, response rate 51.7). | Country, age, seniority, department, professional level, family situation, satisfaction with salary, child care, quality of team work, «overcommitment», disturbances at work, demand, get up before 5:00 a.m., take shifts on short notice, satisfied with change in caregiver, gender, physical burden, weekend work, split shifts. | Cross-sectional. Multivariate analysis. | Nurses who regularly work 10 or 12-hour night shifts had significantly high odds of burnout compared to part-time workers. Nurses who regularly worked 12-hour night shifts also had significantly higher odds of worrying about making mistakes. Part-time workers and nurses on rotating shifts with more than six night shifts per month had significantly higher sick leave than nurses who regularly worked the night shift. The authors concluded that other aspects, such as having to take a shift on short notice, the quality of teamwork and having to get up before 5 a.m. seemed to play a greater role for employee health and safety than the type of shift. |
| Gander, Purnell, Garden, and Woodward (2007) | Number of days at work, weekly overtime, rotation, shift length (under and over 14 hours), night shift. | Sleepiness (ESS).  Self-reported. | Fatigue-related errors.  Self-reported. | Younger doctors in New Zealand, under training and working more than 40 hours per week (N=1366), response rate 63. | Demographic variables | Cross-sectional. Logistical regression | Number of work days in the past two weeks and 14-hour shifts were not significantly related to either sleepiness or errors. Number of hours worked the past two weeks was significantly related to sleepiness (OR 1.54 CI 1.02-2.33), but not errors. Shift rotation (change in type of shift) in both weeks was significantly related to sleepiness (OR 1.81 CI 1.31-2.50), but not errors. Frequent night shifts (>2 nights per week) were related to both sleepiness (OR: 1.53 CI 1.02-2.33) and errors (OR1.37 CI 1.03-1.84). |
| Gander, Merry, Millar, and Weller (2000) | Weekly work hours, night shifts per week, limitations on weekly and daily work hours. |  | Fatigue-related errors | Anaesthetists (N= 236, response rate 70). The selection was divided in two for analysis, according to position (trainees and specialists). | None. | Cross-sectional. Logistical regression. | None of the work hour variables were related to errors caused by fatigue. For specialists there was a correlation between number of nights with work-related disturbances of sleep and errors. Having overstepped self-defined safety boundaries for work hours the past seven days was also significantly related to self-reported errors for specialists. |
| Garden, Robinson, Kappus, Macleod, and Gander (2012) | Two 15-hour shifts (with a nine-hour shift in between). Performance at the start of the first 15-hour shift was compared with performance at the end of the second 15-hour shift. | Sleep. Measured by self-reporting (journal) and wristband (Actiwatch). | Execution of a simulated work situation (appendectomy) in an operating room with medical personnel and researchers as «actors». Evaluated by “blind” expert observers. | Anaesthetists (N= 12). | None. | Each doctor was compared with him-/herself. | The doctors had slept significantly less the last four days after the second 15-hour shift than before the first shift (6.8 hours per day versus 7.3 hours per day). But the difference was small and described by the authors as likely insignificant. 1/3 of the doctors had slept less than 6.5 hours before the second 15-hour shift. There was a non-significant tendency (p=0.06) for the doctors to perform a less adequate check of the equipment after after the second 15-hour shift. There were few differences in execution of duties and the authors commented that the clinical significance of the differences found was unclear. |
| Garland, Roberts, and Graff (2012) | Two types of shift arrangements with intensive care doctors: (1) The same doctor worked 24/7 for 7 days, with on-call duty from home (standard), or (2) The intensive care doctor worked day shift for 7 consecutive days, while another IC doctor worked the night shift. | Burnout (MBI).  Self-reported | The patients’ mortality both in the intensive care unit and at the hospital in general.  Registered data for each unit. | Intensive care doctors in Canada (N=24). | Place of study, workload, source and time of admission, age, gender, co-morbidity, diagnosis and APACHE II score | 32-week crossover experiment. Logistical regression. | Intensive care doctors who worked shifts experienced less burnout than those of standard staffing (p < 0.05). Type of shift/ intensive care arrangements had no significant effect on patient mortality. |
| Gold et al. (1992) | Shifts the past two weeks - night, day/evening with some nights, or rotating shifts compared with day/evening shift. | Sleep quality, dozing off at work.  Self-reported. | Errors and near errors the past year (including medication errors, car accidents, procedural errors and on-the-job injuries).  Self-reported. | Hospital nurses in the USA. (N= 687, response rate 78). | Number of years at the hospital | Cross-sectional. Logistical regression | Nurses on rotating shifts or night shift reported less regular sleep (i.e. sleep at the same time every night) and more disturbed sleep (the difference was shown descriptively, not statistically tested) than daytime workers.  Nurses on night shifts (OR 1.82 CI1.02-3.22) and rotating shifts (OR 2.82 CI 1.79-4.45) reported significantly poorer sleep quality, but no significant difference in fatigue. Nurses on rotating shifts reported increased probability of error (OR 1.97 CI 1.07-3.64) and near error (OR 2.47 CI 1.56-3.89). The difference was not significant for night shift workers (OR 1.88 and 1.55). |
| Gomez-Garcia et al. (2016) | Day shift, night shift and rotating shifts | Burnout (MBI), sleep quality (PSQI), daytime sleepiness | Incorrect medication, pressure sore, fall, various infections, adverse incidents.  Self-reported. | Nurses at seven Spanish hospitals. (N= 635). | None. | Cross-sectional, descriptive, bivariate analysis | No significant difference in burnout between shift types. Night shift workers collectively had poorer sleep quality (measured by several different types of measurement) than day shift workers (p=0.017). There was no significant variation in patient safety between the shift types. |
| Houston and Allt (1997) | Number of hours per week (on average and the past week) | General health (GHQ).  Self-reported. | Medical errors and general everyday cognitive errors.  Self-reported. | Doctors in their first job after studies in England (N=30 response rate 60). | None. | Prospective cohort. One measurement before they start the job and one eight weeks after having started working. | Most employees worked between 60 and 80 hours per week. The work hours did not correlate significantly with health or cognitive errors at any timepoint, or during development from timepoint 1 to timepoint 2. There was a connection between general health and cognitive errors at both timepoints. Cognitive errors were related to medical errors, but general health did not have an independent correlation to medical errors. |
| Jagsi et al. (2005) | Number of hours per week, number of hours per shift, type of rotation. | Chronic fatigue (>days with considerable fatigue the past month).  Self-reported. | Negative incidents (negative consequences for the patient as a result of the medical treatment), errors and near errors.  Self-reported. | Doctors in training in the USA (N=689 response rate 57). | Specialty, seniority. | Cross-sectional. | Among the doctors who reported that the most recent negative incident was the result of an error, 19 percent reported that the cause could be that they had worked too many hours. Included in the same analysis were type of rotation (OR 3.4 p<0.001) and work hours (>80 hours per week) (OR 1.8 p<0.05) related to increased odds of negative incidents. Type of rotation (OR 2.3 p<0.01) and fatigue were related to near errors (OR 1.8 p<0.05). Fatigue was also related to negative incidents when other variables were not controlled for. |
| Kalmbach, Arnedt, Song, Guille, and Sen (2017) | Number of hours per week (more or less than the average). | Depression (PHQ-9), sleep problems and length of sleep (PSQI) (<6 hours).  Self-reported | Medical errors.  Self-reported | Doctors in training in the USA (residents) without depression at the start. (N=1215, response rate 58). | Age, gender and number of work hours. | Prospective cohort. Three measuring points; 1-2 months prior to starting as residents and three and six months into the training. | The doctors’ amount of sleep decreased when they started the training. Having had sleep problems before the training and working 70 hours or more per week were significantly related to less sleep the first months of the training. Both having sleep problems and number of work hours had independent correlations with the odds of depression after three months (in a multiple logistical analysis). Further analysis showed that both length of sleep and sleep problems had independent correlations with the odds of depression, controlled for age, gender and work hours; however, in this analysis the connection between work hours and depression was not provided by the authors.  For those who were depressed at three months, sleep length was related to still having depression at six months, but number of work hours was not related.  Each hour less of sleep was related to a 27 percent increase in the odds of medical error. Broken down by measuring point, the increase was significant after three months but not after six months. Working more tha 70 hours per week and being depressed were significantly related to increased risk of error after three and six months. The researchers found the highest risk of error among the chronically depressed (those who were depressed at both three and six months). |
| Kaneita and Ohida (2011) | Work hours per week, days on call and overnight work | Length of sleep, sleep deprivation, insomnia | Medical errors that were noticed before or after, but did not had serious consequences | Members of the Japan Medical Association (N= 3486, response rate 77). | Gender, age, hiring status and institution | Cross-sectional, logistical regression | Higher probability of medical incident among doctors who had worked long days (OR 1.72 for >10 hours), had worked overnight or on call (two-three times OR 1.54, CI 1.09 – 2.16; four to seven times OR 1.49, 1.06-2.08; but not eight or more times) per month, and among those who were sleep deprived (OR 1.65, 1.33-2.04) or suffered from insomnia (OR 1.45, 1.16-1.82). |
| Keshk and Abd El-Moneem (2012) | Weekly work hours.  Self-reported | Fatigue (subjective feeling of concentration, motivation and physical fatigue).  Self-reported | Errors in preparation and administration of medicines. Each nurse was observed over a given period and errors were coded. | Nurses in Cairo. (Two selections with N=29 and N=20). | None. | Cross-sectional. | There was no significant correlation between work hours and errors in any of the selections (p = 0.3 to 0.7). There was a significant correlation between higher number of weekly work hours and greater physical fatigue in both selections (p>0.05). There was also a non-significant connection between higher number of work hours and concentration fatigue in one of the selections (p=0.051). Greater concentration fatigue was significantly related to more errors in preparation and administration of medicines in one of the selections and more errors in the preparation of medicines in the other. Motivation fatigue was significantly related to more errors in the administration of medicines for one of the selections. |
| Kunaviktikul et al. (2015) | Work hours per week (number of hours over 40 hours per week) | Emotional fatigue, depersonalization (two components of burnout-MBI).  Self-reporting | Incorrect patient identification, pressure sores, communication errors, patient complaints).  Self-reporting | Nurses in hospitals throughout Thailand (N= 1524) | Age, gender and education | Cross-sectional. Descriptive statistics, Spearman’s correlation analysis, logistical regression | Long work weeks related to increased risk of emotional fatigue (b 0.085, p 0.001) and depersonalization (b 0.08, p 0.002). There were also significantly higher odds of negative patient outcomes the higher the number of hours the nurses worked per week. The highest collective risk of negative outcomes was when the work week was 65-72 hours (OR 1.85 (CI 1.24-2.77). |
| Lobo, Ploeg, Fisher, Peachey, and Akhtar-Danesh (2017) | Overtime – particularly staying on at the end of the shift and missing breaks. | Nurses interviewed about the consequences of overtime | Nurses interviewed about the consequences of overtime | Nurses in hospitals in Canada (N=28). |  | Qualitative | The nurses experienced physical consequences of overtime; particularly physical pain such as neck and shoulder pain, fatigue, hunger and dehydration, and increased susceptibility to illness. The nurses also emphasized that safety was reduced for both themselves and the patients – when the overtime led to less sleep and busier periods, they became more fatigued and less able to perform the job correctly. As examples, they claimed that when they were tired, they were less inclined to follow procedures (e.g. to prevent infections), they experienced reduced level of skills and increased probability of making a mistake. The nurses also reported other negative consequences for patient treatment such as poorer communication and a more task-oriented rather than patient-oriented focus. |
| McCawley, Cyna, Prineas, and Tan (2017) |  |  | Were asked to cite the medication error they remembered best. | Anaesthetists in Australia and New Zealand. (N=295 response rate 29.8). |  | Descriptive | The episode had occurred during normal work hours in 71 percent of the cases, and most often (36 percent) between four and 12 hours into the shift. In more than 80 percent of the cases the error had not led to patient harm. The doctors experienced a spectrum of feelings afterwards, from embarrassment (62 percent) to disappointment, irritation and guilt (41 percent). 14 percent claimed that the episode had affected their sleep, and 18 percent said it had affected their mood. 1 percent reported having sought professional help after the incident. |
| McIntyre, Winfield, Te, and Crook (2010) | Reduction in work hours from 56 to 48 hours per week, and rest requirement (without increasing staffing). Reduction was due to introduction of the European directive. | Sick leave was measured the year prior to and the year after the reform. Measured as number of doctors on sick leave, number of absences, number of absences per doctor, total number of days absent, days per absence and number of doctors with more than one absence per year. Data was taken from the hospital register. | Mortality while staying at the hospital, readmissions, length of stay. Register data | Younger doctors in a hospital in Great Britain (varying number through the period 58 and 61), their sick leaves, admissions and readmissions, mortality. | The patients’ age | Retrospective observational survey, before and after. | The number of doctors on sick leave increased 90 percent (p<0.001), while the number of sick leave days per absence and all measured variables for patient outcomes were unchanged. |
| Olds and Clarke (2010) | Weekly work hours (>40 hours per week or ≤40 hours per week), and volunteer overtime | Needle sticks, work-related employee injuries.  Self-reported | Incorrect medicine or dosage, patient outcomes with injury, and infections contracted at the hospital.  Self-reported. | Nurses in 188 hospitals in the USA (N = 11 516, response rate 52). | Gender, workplace, age, work experience, education, hospital staffing, children/dependents at home, permanently employed, union membership. | Cross-sectional. Logistical regression. | All negative outcomes were more common among nurses who worked more than 40 hours per week; incorrect medicine or dosage (OR 1.28 CI 1.10-1.49), patient outcomes with injury (OR 1.17 CI 1.02-1.36), and infections contracted at the hospital (OR 1.14 CI 1.02-1.28), needle sticks (OR 1.28 CI 1.08-1.52), and work-related employee injuries (OR 1.25 CI 1.11-1.40).  Similar findings were also reported for paid volunteer overtime. |
| Parshuram et al. (2015) | Shifts of 12-16 and 24 hours. | Sleepiness (SSS), mental symptoms, burnout (MBI) | Adverse incidents, mortality | Doctors in Canadian hospitals (N= 47), admissions (N=971), patient days (N=5 894) and surveys of intensive care staff (N= 452 response rate 47,1) and other doctors and nurses (N=217, response rate 75.3). | APACHE score | Experimental design, randomized distribution of shifts | No effect from different shift lengths on adverse incidents, burnout or doctors’ sleepiness. The mortality rates were also equal. Significantly more frequent mental symptoms among the doctors who worked 24-hour shifts (p>0.05). |
| Qureshi et al. (2015) | Work hours (>70 hours per week). On call at night (>2 times a week) | Burnout (MBI) and depression (PRIME MD). Self-reported | Medical errors.  Self-reported | Plastic surgeons in the USA (N=1691, response rate 28.5). | Specialty, compensation, salary, position, workload, non-clinical activities, work experience, civil status, children, age. | Cross-sectional. Logistical regression | Working more than 70 hours per week (OR 2.42 p<0.001) and being on call more than two nights per week (OR 1.95 p<0.001) were significantly related to increased risk of burnout. In the analysis that looked at outcomes of burnout, burnout increased the odds of depression (OR 1.21 p<0.05) and making medical errors (OR 1.89 p<0.001). |
| Ramadan and Al-Saleh (2014) | Shift length, work hours per week. | Sleep deprivation (PSQI). Self-reported | Medical errors.  Self-reported | Nurses in Saudi Arabia who worked night shift (N=138). | Not provided. | Cross-sectional. | 19 percent of the selection reported having sleep deprivation.  Nurses with sleep deprivation reported more medical errors than nurses without sleep deprivation. Independent analysis of 8-hour shifts, 10-hour shifts, and 12-hour shifts showed significant correlations between sleep deprivation and more frequent errors for all shift lengths.  Number of hours worked per week, but not the number of hours slept per week, was also associated with the number of reported errors (B 0.68 p<0.001). |
| Scott, Rogers, Hwang, and Zhang (2006) | Shift length (≤8.5, 8.5-12.5, ≥12.5) | Sleepiness.  Self-reported (diary). | Errors and near errors. The most common errors were in administration of medicines.  Self-reported. | Nurses in the USA (N=502 response rate 18). | Demographic variables and hospital characteristics. | Diary study. GEE | The results showed that, in most cases, the nurses worked longer shifts than planned. They worked 41 hours per week on average. When the nurses worked 12.5 hours or more the odds increased for making a mistake (OR 1.94, p> 0.05), being sleepy on the job (OR 1.5 p<0.01) and dozing off on the job (OR 2.4 p<0.05). The authors also reported that working more than 40 hours per week was related to errors and near errors, but that there was no significant connection between struggling to stay awake and increased risk of error. The last analyses were not shown. |
| Scott, Arslanian-Engoren, and Engoren (2014) | Shift length (12 hours) and shift type. | Sleep (Rosenkind), fatigue, sleep quality (PSQI) and sleepiness (ESS).  Self-reported. | Have regretted a clinical decision made while sleepy.  Self-reported. | Nurses in the USA (N=546 response rate 22). | Gender, satisfaction with clinical decisions. | Cross-sectional. | Higher level of daytime sleepiness (b 0.08 p<0,05), sleep deprivation (b 0.34 p<0.05) and 12-hour shifts (b 0.89 p<0.05) were associated with higher odds of individual regret, but only 12-hour shifts associated with regret were included in the same model. The authors concluded that 12-hour shifts could lead to sleepiness, but that it likely was not the sleepiness that led to the incidents the nurses regretted. Fatigue, sleep quality and night work were also associated with regretting a decision, but this was no longer significant in the logistical regression with control variables. |
| Seki and Yamazaki (2006) | Day shift, evening shift and night shift | Length of sleep and fatigue (measured prior to the start of the shift).  Self-reported | Near errors.  Self-reported | Japanese nurses (N=88, response rate 97.8) followed for 525 consecutive days. | Type of department | Longitudi-nal.  Logistical regression with GEE | Fatigue did not lead to a significant increase in near errors for any of the shift types, while little sleep led to increased number of errors on the night shift: OR 1.242 (1.049-1.470). Delays due to busy periods showed a clearer impact on error percentage on both the day and night shifts. |
| Stimpfel, Lake, Barton, Gorman, and Aiken (2013) | Shift length (8, 12 or >13 hours) | Burnout (MBI).  Self-reported | Infections, frequent central line infections, frequent urinary tract infections, general safety in the unit.  Self-reported | Nurses in pediatric units at over 300 hospitals in the USA (N=3710). | Age, gender, intensive care unit, staffing, environment, hospital characteristics (e.g. number of beds), job satisfaction, intention to quit, patient complaints, quality of care. | Cross-sectional.  Logistical regression. | The results showed significantly higher burnout with >13-hour shifts (OR 2.73 CI 1.86, 4.00), increased risk of central line infection (OR 2.54 CI 1.50- 4.32), low general safety evaluation (OR 3.14 CI 2.11-4.68) and increased risk that information would be lost during shift change (OR 2.10 CI 1.40-3.15), but a non-significant increase in urinary tract infections (OR 1.84 CI .78-4.33) |
| Suzuki et al. (2004)  This is the same selection as for Suzuki 2005 | Night shift and irregular shifts | Mental health (GHQ), sleep quality.  Self-reported. | Accidents the past 12 months (medication errors, use of medical equipment, incorrect patient identification, needle sticks).  Self-reported. | Japanese nurses at eight different hospitals (N= 4297, response rate 94). | Not clearly specified, included with stepwise elimination | Cross-sectional survey, logistical regression | Risk of accidents increased with shift work (OR 1.78, 1.35-2.34) and reduced mental health (OR 1.55, 1.32-1.82). Each factor had significant effect when controlled for the other |
| Suzuki, Ohida, Kaneita, Yokoyama, and Uchiyama (2005)  This is the same selection as for Suzuki 2004 | Night shift and irregular shifts | Daytime sleepiness (PSQI), inadequate sleep, alcohol or medication to fall asleep | Accidents the past 12 months: incorrect medication, use of medical equipment, needle sticks | Japanese nurses at eight different hospitals (N= 4297, response rate 94). | Not clearly specified, included with stepwise elimination | Cross-sectional survey, logistical regression | Risk of incorrect medication increased with shift work (OR 1.78 (1.35-2.34), and incorrect use of medical equipment with considerable daytime sleepiness (OR 1.27, 1.09-1.49) |
| Weaver, Stutzman, Supnet, and Olson (2016) | Day or night shift (12-hour shifts) | Sleep quality and quantity (PSQI).  Self-reported and measured with wristband | Errors at the end of the shift (minor, moderate and serious).  Self-reported | Nurses in the emergency department at an American hospital (N= 30). | Experience as a nurse | Prospective cohort.  The wristband measured sleep prior to a 12-hour shift and the nurses reported errors after the shift. | Those who worked day shift spent more time in bed but did not sleep significantly more hours per night than those who worked the night shift. Those who worked the day shift were more inclined to awaken again after having fallen asleep (lower sleep quality) No significant difference in sleep quality or errors between the day and night shifts.  Sleep quality measured in number of hours did not impact the reported errors, but reduced sleep quality (hours in bed/hours asleep) was tied to increased frequency of self-perceived minor errors (p= 0.02). |
| Wen et al. (2016) | Work hours per week | Burnout (MBI, Chinese version).  Self-reported | Errors in the form of patient injury and incorrect medication, delayed treatment, incorrectly filled out journal.  Self-reported. | Doctors from hospitals in 10 Chinese provinces (N= 1537, response rate 89.3). | Not clearly specified, included with stepwise elimination | Cross-sectional survey, logistical stepwise regression | The risk of error increased with 60 work hours or more per week (OR 1.65, 1.22-2.22) and with considerable burnout (OR 2.28, 1.63-3.17). Each factor had significant effect when controlled for the other |

CES-D. Center for Epidemiologic Studies Depression Scale

ESS. Epworth Sleepiness Scale

GHQ-28. General Health Questionnaire, 28 spm. 12 har 12 spm etc.

HANDS: Harvard National Depression Screening Day Scale

KSS. Karolinska Sleepiness Scale

MBI. Maslach Burnout Inventory.

PHQ-9. The Patient Health Questionnaire. 9 spm.

PRIME MD. Primary Care Evaluation of Mental Disorders.

PSQI. Pittsburgh Sleep Quality Index

Rosenkind. Formula for calculating sleep debt.

SDQ. Sleep Disorder Questionnaire (Zomer et al 1985).

SSS. Stanford Sleepiness Scale.

Admi, H., Tzischinsky, O., Epstein, R., Herer, P., & Lavie, P. (2008). Shift work in nursing: is it really a risk factor for nurses' health and patients' safety? *Nursing Economics, 26*(4), 250-257.

Ali, N. A., Hammersley, J., Hoffmann, S. P., O'Brien, J. M., Jr., Phillips, G. S., Rashkin, M., . . . Midwest Critical Care, C. (2011). Continuity of care in intensive care units: a cluster-randomized trial of intensivist staffing. *American Journal of Respiratory & Critical Care Medicine, 184*(7), 803-808. doi:<https://dx.doi.org/10.1164/rccm.201103-0555OC>

Amirian, I., A. K. Danielsen, & Rosenberg, J. (2013). Perception of Fatigue Among Surgeons During Night Shifts *Ann R Coll Surg Engl, 95*(7), (Suppl). doi:<https://doi.org/10.1308/147363513X13690603821026>

Amirian, I., Andersen, L. T., Rosenberg, J., & Gögenur, I. (2014). Laparoscopic Skills and Cognitive Function are not Affected in Surgeons During a Night Shift. *Journal of Surgical Education, 71*(4), 543-550. doi:<https://doi.org/10.1016/j.jsurg.2013.12.007>

Arakawa, C., Kanoya, Y., & Sato, C. (2011). Factors Contributing to Medical Errors and Incidents among Hospital Nurses -Nurses' Health, Quality of Life, and Workplace Predict Medical Errors and Incidents. *Industrial Health, 49*(3), 381-388. doi:10.2486/indhealth.MS968

Arimura, M., Imai, M., Okawa, M., Fujimura, T., & Yamada, N. (2010). Sleep, mental health status, and medical errors among hospital nurses in Japan. *Industrial Health, 48*(6), 811-817.

Arzalier-Daret, S., Buleon, C., Bocca, M. L., Denise, P., Gerard, J. L., & Hanouz, J. L. (2017). Effect of sleep deprivation after a night shift duty on simulated crisis management by residents in anaesthesia. A randomised crossover study. *Anaesthesia Critical Care & Pain Medicine, 04*, 04. doi:<https://dx.doi.org/10.1016/j.accpm.2017.05.010>

Asaoka, S., Aritake, S., Komada, Y., Ozaki, A., Odagiri, Y., Inoue, S., . . . Inoue, Y. (2013). Factors associated with shift work disorder in nurses working with rapid-rotation schedules in Japan: the nurses' sleep health project. *Chronobiology International, 30*(4), 628-636. doi:<https://dx.doi.org/10.3109/07420528.2012.762010>

Bae, S. H. (2013). Presence of nurse mandatory overtime regulations and nurse and patient outcomes. *Nursing Economics, 31*(2), 59-68, 89; quiz 69.

Balch, C. M., Oreskovich, M. R., Dyrbye, L. N., Colaiano, J. M., Satele, D. V., Sloan, J. A., & Shanafelt, T. D. (2011). Personal Consequences of Malpractice Lawsuits on American Surgeons. *Journal of the American College of Surgeons, 213*(5), 657-667. doi:10.1016/j.jamcollsurg.2011.08.005

Baldwin, D. C., Jr., & Daugherty, S. R. (2004). Sleep deprivation and fatigue in residency training: results of a national survey of first- and second-year residents. *Sleep, 27*(2), 217-223.

Baldwin, D. C., Jr., Daugherty, S. R., Tsai, R., & Scotti, M. J., Jr. (2003). A national survey of residents' self-reported work hours: thinking beyond specialty. *Academic Medicine, 78*(11), 1154-1163.

Barger, L. K., Ayas, N. T., Cade, B. E., Cronin, J. W., Rosner, B., Speizer, F. E., & Czeisler, C. A. (2006). Impact of extended-duration shifts on medical errors, adverse events, and attentional failures. *PLoS Medicine / Public Library of Science, 3*(12), e487.

Bilimoria, K. Y., Chung, J. W., Hedges, L. V., Dahlke, A. R., Love, R., Cohen, M. E., . . . Lewis, F. R. (2016). National Cluster-Randomized Trial of Duty-Hour Flexibility in Surgical Training. *New England Journal of Medicine, 374*(8), 713-727. doi:<https://dx.doi.org/10.1056/NEJMoa1515724>

Cammu, H., & Haentjens, P. (2012). Perceptions of fatigue - and perceived consequences - among Flemish obstetricians-gynaecologists: a survey. *European Journal of Contraception & Reproductive Health Care, 17*(4), 314-320. doi:<https://dx.doi.org/10.3109/13625187.2012.672664>

Cappuccio, F. P., Bakewell, A., Taggart, F. M., Ward, G., Ji, C., Sullivan, J. P., . . . Warwick, E. W. G. (2009). Implementing a 48 h EWTD-compliant rota for junior doctors in the UK does not compromise patients' safety: assessor-blind pilot comparison. *Qjm, 102*(4), 271-282. doi:<https://dx.doi.org/10.1093/qjmed/hcp004>

Chen, I., Vorona, R., Chiu, R., & Ware, J. C. (2008). A survey of subjective sleepiness and consequences in attending physicians. *Behavioral Sleep Medicine, 6*(1), 1-15. doi:<https://dx.doi.org/10.1080/15402000701796023>

Chen, K. Y., Yang, C. M., Lien, C. H., Chiou, H. Y., Lin, M. R., Chang, H. R., & Chiu, W. T. (2013). Burnout, job satisfaction, and medical malpractice among physicians. *International Journal of Medical Sciences, 10*(11), 1471-1478. doi:<https://dx.doi.org/10.7150/ijms.6743>

de Oliveira, G. S., Jr., Chang, R., Fitzgerald, P. C., Almeida, M. D., Castro-Alves, L. S., Ahmad, S., & McCarthy, R. J. (2013). The prevalence of burnout and depression and their association with adherence to safety and practice standards: a survey of United States anesthesiology trainees. *Anesthesia & Analgesia, 117*(1), 182-193. doi:<https://dx.doi.org/10.1213/ANE.0b013e3182917da9>

Dean, G. E., Scott, L. D., & Rogers, A. E. (2006). Infants at risk: when nurse fatigue jeopardizes quality care. *Advances in Neonatal Care, 6*(3), 120-126.

Domen, R., Connelly, C. D., & Spence, D. (2015). Call-shift fatigue and use of countermeasures and avoidance strategies by certified registered nurse anesthetists: a national survey. *AANA Journal, 83*(2), 123-131.

Dorrian, J., Lamond, N., van den Heuvel, C., Pincombe, J., Rogers, A. E., & Dawson, D. (2006). A pilot study of the safety implications of Australian nurses' sleep and work hours. *Chronobiology International, 23*(6), 1149-1163.

Dorrian, J., Tolley, C., Lamond, N., van den Heuvel, C., Pincombe, J., Rogers, A. E., & Drew, D. (2008). Sleep and errors in a group of Australian hospital nurses at work and during the commute. *Applied Ergonomics, 39*(5), 605-613. doi:<https://dx.doi.org/10.1016/j.apergo.2008.01.012>

Eastridge, B. J., Hamilton, E. C., O'Keefe, G. E., Rege, R. V., Valentine, R. J., Jones, D. J., . . . Thal, E. R. (2003). Effect of sleep deprivation on the performance of simulated laparoscopic surgical skill. *American Journal of Surgery, 186*(2), 169-174.

Estryn-Behar, M., Van der Heijden, B., & Grp, N. S. (2012). Effects of extended work shifts on employee fatigue, health, satisfaction, work/family balance, and patient safety. *Work-a Journal of Prevention Assessment & Rehabilitation, 41*, 4283-4290. doi:10.3233/wor-2012-0724-4283

Gander, P., Purnell, H., Garden, A., & Woodward, A. (2007). Work patterns and fatigue-related risk among junior doctors. *Occupational & Environmental Medicine, 64*(11), 733-738.

Gander, P. H., Merry, A., Millar, M. M., & Weller, J. (2000). Hours of work and fatigue-related error: a survey of New Zealand anaesthetists. *Anaesthesia & Intensive Care, 28*(2), 178-183.

Garden, A. L., Robinson, B. J., Kappus, L. J., Macleod, I., & Gander, P. H. (2012). Fifteen-hour day shifts have little effect on the performance of taskwork by anaesthesia trainees during uncomplicated clinical simulation. *Anaesthesia and Intensive Care, 40*(6), 1028-1034.

Garland, A., Roberts, D., & Graff, L. (2012). Twenty-four-hour intensivist presence: a pilot study of effects on intensive care unit patients, families, doctors, and nurses. *American Journal of Respiratory & Critical Care Medicine, 185*(7), 738-743. doi:<https://dx.doi.org/10.1164/rccm.201109-1734OC>

Gold, D. R., Rogacz, S., Bock, N., Tosteson, T. D., Baum, T. M., Speizer, F. E., & Czeisler, C. A. (1992). Rotating shift work, sleep, and accidents related to sleepiness in hospital nurses. *American Journal of Public Health, 82*(7), 1011-1014.

Gomez-Garcia, T., Ruzafa-Martinez, M., Fuentelsaz-Gallego, C., Madrid, J. A., Rol, M. A., Martinez-Madrid, M. J., . . . Group, R. (2016). Nurses' sleep quality, work environment and quality of care in the Spanish National Health System: observational study among different shifts. *BMJ Open, 6*(8), e012073. doi:<https://dx.doi.org/10.1136/bmjopen-2016-012073>

Houston, D. M., & Allt, S. K. (1997). Psychological distress and error making among junior house officers. *British Journal of Health Psychology, 2*(Part 2), 141-151. doi:<http://dx.doi.org/10.1111/j.2044-8287.1997.tb00530.x>

Jagsi, R., Kitch, B. T., Weinstein, D. F., Campbell, E. G., Hutter, M., & Weissman, J. S. (2005). Residents report on adverse events and their causes. *Archives of Internal Medicine, 165*(22), 2607-2613.

Kalmbach, D. A., Arnedt, J. T., Song, P. X., Guille, C., & Sen, S. (2017). Sleep Disturbance and Short Sleep as Risk Factors for Depression and Perceived Medical Errors in First-Year Residents. *Sleep, 40*(3), 01. doi:<https://dx.doi.org/10.1093/sleep/zsw073>

Kaneita, Y., & Ohida, T. (2011). Association of current work and sleep situations with excessive daytime sleepiness and medical incidents among Japanese physicians. *Journal of Clinical Sleep Medicine, 7*(5), 512-522. doi:<https://dx.doi.org/10.5664/JCSM.1322>

Keshk, L. I., & Abd El-Moneem, D. S. (2012). Effect of Nurses' Work Hours and Fatigue on Occurrence of Medication Errors in ICU and Medical Oncology Unit -Cairo University. *Life Science Journal-Acta Zhengzhou University Overseas Edition, 9*(3), 347-355.

Kunaviktikul, W., Wichaikhum, O., Nantsupawat, A., Nantsupawat, R., Chontawan, R., Klunklin, A., . . . Sirakamon, S. (2015). Nurses' extended work hours: Patient, nurse and organizational outcomes. *International Nursing Review, 62*(3), 386-393. doi:<https://dx.doi.org/10.1111/inr.12195>

Lobo, V. M., Ploeg, J., Fisher, A., Peachey, G., & Akhtar-Danesh, N. (2017). Critical care nurses' perceptions of the outcomes of working overtime in Canada. *Nursing Outlook, 65*(4), 400-410. doi:10.1016/j.outlook.2016.12.009

McCawley, D., Cyna, A. M., Prineas, S., & Tan, S. (2017). A survey of the sequelae of memorable anaesthetic drug errors from the anaesthetist's perspective. *Anaesthesia & Intensive Care, 45*(5), 624-630.

McIntyre, H. F., Winfield, S., Te, H. S., & Crook, D. (2010). Implementation of the European Working Time Directive in an NHS trust: impact on patient care and junior doctor welfare. *Clinical Medicine, 10*(2), 134-137.

Olds, D. M., & Clarke, S. P. (2010). The effect of work hours on adverse events and errors in health care. *Journal of Safety Research, 41*(2), 153-162. doi:<https://dx.doi.org/10.1016/j.jsr.2010.02.002>

Parshuram, C. S., Amaral, A. C., Ferguson, N. D., Baker, G. R., Etchells, E. E., Flintoft, V., . . . Canadian Critical Care Trials, G. (2015). Patient safety, resident well-being and continuity of care with different resident duty schedules in the intensive care unit: a randomized trial. *CMAJ Canadian Medical Association Journal, 187*(5), 321-329. doi:<https://dx.doi.org/10.1503/cmaj.140752>

Qureshi, H. A., Rawlani, R., Mioton, L. M., Dumanian, G. A., Kim, J. Y., & Rawlani, V. (2015). Burnout phenomenon in U.S. plastic surgeons: risk factors and impact on quality of life. *Plastic & Reconstructive Surgery, 135*(2), 619-626. doi:<https://dx.doi.org/10.1097/PRS.0000000000000855>

Ramadan, M. Z., & Al-Saleh, K. S. (2014). The association of sleep deprivation on the occurrence of errors by nurses who work the night shift. *Current Health Sciences Journal, 40*(2), 97-103. doi:<https://dx.doi.org/10.12865/CHSJ.40.02.03>

Scott, L. D., Arslanian-Engoren, C., & Engoren, M. C. (2014). Association of sleep and fatigue with decision regret among critical care nurses. *American Journal of Critical Care, 23*(1), 13-23. doi:<https://dx.doi.org/10.4037/ajcc2014191>

Scott, L. D., Rogers, A. E., Hwang, W. T., & Zhang, Y. (2006). Effects of critical care nurses' work hours on vigilance and patients' safety. *American Journal of Critical Care, 15*(1), 30-37.

Seki, Y., & Yamazaki, Y. (2006). Effects of working conditions on intravenous medication errors in a Japanese hospital. *Journal of Nursing Management, 14*(2), 128-139.

Stimpfel, A. W., Lake, E. T., Barton, S., Gorman, K. C., & Aiken, L. H. (2013). How differing shift lengths relate to quality outcomes in pediatrics. *Journal of Nursing Administration, 43*(2), 95-100. doi:<https://dx.doi.org/10.1097/NNA.0b013e31827f2244>

Suzuki, K., Ohida, T., Kaneita, Y., Yokoyama, E., Miyake, T., Harano, S., . . . Uchiyama, M. (2004). Mental health status, shift work, and occupational accidents among hospital nurses in Japan. *Journal of Occupational Health, 46*(6), 448-454.

Suzuki, K., Ohida, T., Kaneita, Y., Yokoyama, E., & Uchiyama, M. (2005). Daytime sleepiness, sleep habits and occupational accidents among hospital nurses. *Journal of Advanced Nursing, 52*(4), 445-453.

Weaver, A. L., Stutzman, S. E., Supnet, C., & Olson, D. M. (2016). Sleep quality, but not quantity, is associated with self-perceived minor error rates among emergency department nurses. *International emergency nursing, 25*, 48-52. doi:<https://dx.doi.org/10.1016/j.ienj.2015.08.003>

Wen, J., Cheng, Y., Hu, X., Yuan, P., Hao, T., & Shi, Y. (2016). Workload, burnout, and medical mistakes among physicians in China: A cross-sectional study. *Bioscience Trends, 10*(1), 27-33. doi:<https://dx.doi.org/10.5582/bst.2015.01175>
